# Unpacking the growth of global agricultural greenhouse gas emissions

**DOI:** 10.1126/sciadv.aeb8653

**Published:** 2026-01-16

**Authors:** Ariel Ortiz-Bobea, Simone Pieralli

**Affiliations:** ^1^ Charles H. Dyson School of Applied Economics and Management and Jeb E. Brooks School of Public Policy, Cornell University, Ithaca, NY, USA.; ^2^ European Commission, Joint Research Centre (JRC), Seville, Spain.

## Abstract

Agriculture, forestry, and other land use contribute about a fifth of total anthropogenic greenhouse gas (GHG) emissions. Mitigation efforts have emphasized “decoupling” that sustains production while lowering emissions per unit of output. However, the underlying decoupling mechanisms have not been fully characterized. We rely on a mathematical identity to decompose agricultural GHG emission growth (ΔE) into three parts: output (ΔY), emissions per unit of input (ΔE/X), and output per unit of input (ΔY/X) or total factor productivity (TFP). We then rely on official country-level data to quantify the historical contribution of these components. Over 1961 to 2021, we find that TFP growth—which captures the sector’s ability to produce more output per unit of measured input—has consistently remained one of the main sources of GHG emission reduction within farms. Further decomposition reveals a key role for rising land productivity in reducing emission intensity.

## Teaser

Productivity growth is the main force slowing growth of agricultural GHG emissions.

## INTRODUCTION

Food systems are an important contributor to global greenhouse gas (GHG) emissions (*1*). These emissions are typically categorized by their physical source and are largely driven by land use change (51%), enteric fermentation and manure management (26%), managed soils and pastures (11%), and rice cultivation (8%) (*2*). Various scenarios suggest that limiting global warming to 2° to 3°C will require substantial GHG emission reductions from the agricultural sector (*2*–*4*).

Debates on reducing agricultural GHG emissions have largely focused on “decoupling” pathways, which aim to reduce emissions while sustaining growth in production (*5*, *6*). Although total emissions from agriculture continue to rise, output emission intensity, defined as emissions per unit of agricultural product, has declined (*7*). These reductions are necessary for decoupling, yet recent evidence suggests that progress is slowing (*8*, *9*).

The agricultural sector can reduce emissions via different mechanisms. Conceptually, emissions are an undesired by-product of production that cannot be freely eliminated or disposed of (*10*). Producers can either reallocate inputs to lower emissions within existing technological constraints, albeit at the cost of sacrificing output, or adopt a new technology that more efficiently converts inputs into desired outputs, thereby reducing the undesirable by-product. Accordingly, decoupling strategies have been roughly classified into those that lower emissions within the existing technology [e.g., (*11*, *12*)] and those that intensify production or raise agricultural productivity [e.g., (*13*–*15*)]. The former emphasizes abatement costs and compensatory payments to encourage farmer adoption of best management practices, while the latter focuses on the costs of research and development (R&D) to achieve productivity gains.

Previous work suggests that raising agricultural productivity could be a cost-effective strategy to reduce emissions. Burney *et al.* (*13*) quantified both the contribution and cost of yield growth in lowering historical GHG emissions. More recent research has evaluated the cost-effectiveness of alternative strategies. For example, Fuglie *et al.* (*16*) models future scenarios comparing investments in productivity-enhancing R&D with the costs of implementing GHG mitigation policies and finds R&D to be the more effective pathway. Fuglie *et al.* (*17*) reaches a similar conclusion in a review exploring how gains in agricultural productivity have helped lower both total and land-use–related GHG emissions. In addition, some work finds that climate-smart policies that encourage soil carbon sequestration may be less effective, as they might induce higher emissions via cropland expansion (*18*).

While existing research has shed light on the role of agricultural productivity gains in reducing emissions, relatively less attention has been devoted to the role of inputs beyond land. Previous work has explored the land-sparing effects of agricultural productivity gains [e.g., (*19*)] or the decomposition of the drivers of land-use–related emissions [e.g., (*20*, *21*). However, agriculture relies on more than land alone. How changes in the levels and productivity of other inputs—such as labor, capital, and materials—shape decoupling remains poorly understood.

The evolution of input emission intensity is fundamentally linked to the process of agricultural development and the emerging patterns of input substitution (*22*). In early stages of development, agriculture depends largely on land and labor—of which only land is arguably directly tied to GHG emissions. As countries develop, technical change may increase the productivity of all or some inputs depending on various fundamentals (*23*). Over time, we see modern inputs such as fertilizers, improved seeds, irrigation, and machinery substitute for land and labor, reflecting either land-saving or labor-saving technological change. Land-scarce economies tend to adopt land-saving, input-intensive paths that may reduce emission intensity. In contrast, land-abundant economies tend to adopt labor-saving pathways such as mechanization, which potentially increase (or at least do not reduce) emission intensity. This helps anticipate how input use and partial productivity growth can jointly contribute to agricultural emissions.

This gap matters because the same decline in output emission intensity, a very common metric, can arise through different mechanisms. Clarifying the relative importance of these channels is essential to explain historical decoupling trends and to guide future strategies. Our study builds on recent advances in the measurement of aggregate productivity that accounts for undesired by-products (*24*–*26*) to provide a more comprehensive account of the drivers of agricultural decoupling.

We decompose the evolution of agricultural GHG emissions to identify and quantify its underlying drivers. We propose a mathematical identity that incorporates agricultural inputs and decomposes the growth of GHG emissions into three parts: output (ΔY), emissions per unit of input or input emission intensity (ΔE/X), and output per unit of input or total factor productivity (TFP) (ΔY/X). We then apply this decomposition to official global datasets of country-level agricultural production, inputs, and GHG emissions (*27*, *28*). We further decompose these channels to better appreciate the distinct role of specific inputs (i.e., capital, labor, materials, and land) over time and across regions. We describe our data sources in greater detail in the Supplementary Materials.

### An expanded decomposition of GHG emission growth that incorporates aggregate inputs

Much of the work describing the evolution of agricultural production and its GHG emissions focuses on decoupling, which consists of increasing production while reducing emissions. The starting point is often an identity of the form E=Y×E/Y where E is GHG emission and Y is aggregate agricultural output. Unpacking the coevolution of economic and environmental indicators has often relied on similar relationships as a basis for decompositions [e.g., (*20*, *21*, *29*, *30*)]. Taking logs and first differences on both sides of this expression leads to a simple decomposition

ΔE=ΔY+Δ(E/Y) (1)

Emission growth ΔE can be decomposed as the sum of output growth ΔY and growth of emissions per unit of output Δ(E/Y) or output emission intensity. One can reduce emissions if the decline in E/Y outpaces the growth of Y. Decoupling essentially requires a decline in E/Y, by definition. However, a reduction in E/Y does not shed light on the drivers of decoupling.

Following (*25*), we expand this identity by introducing an index of agricultural inputs, X. This index aggregates various types of inputs including land, capital, labor, and material inputs based on their cost shares (see the Supplementary Materials and table S1). We explore specific input trends later. Specifically, we recognize that output emission intensity E/Y can also be expressed as (E/X)×(X/Y) or (E/X)/(Y/X), which is the ratio of input emission intensity (E/X) and TFP (Y/X). TFP is a familiar metric that is commonly used in economics to track productivity of sectors in the economy (*31*, *32*). We expand Eq. 1 as

$$\underset{\begin{array}{c} \text{Growth in}\\ \text{Emissions} \end{array}}{\underbrace{\Delta E}}=\underset{\begin{array}{c} \text{Growth in}\\ \text{output} \end{array}}{\underbrace{\Delta Y}}+\underset{\begin{array}{c} \text{Growth in}\\ \text{input emission}\\ \text{intensity} \end{array}}{\underbrace{\Delta \left(E/X\right)}}-\underset{\begin{array}{c} \text{Growth in}\\ \text{total factor}\\ \text{productivity} \end{array}}{\underbrace{\Delta \left(Y/X\right)}}$$ (2)

We unpack emission growth into three components: the growth of output, the growth in input emission intensity, and the growth in TFP. This exposes three channels through which emissions may change. Obviously, emissions could be reduced by changing the level and composition of agricultural production (ΔY). Reducing output level is undesirable, as it would undermine food security and put upward pressure on food prices. Changing the composition of output without demand-side dietary shifts away from ruminant livestock may be unrealistic (*33*). Interesting scenarios arise from the decomposition of Δ(E/Y) into Δ(E/X) and Δ(Y/X). Output emission intensity growth, Δ(E/Y), can decrease by either decreasing input emission intensity growth, Δ(E/X), or boosting TFP growth, Δ(Y/X). The proposed decomposition in Eq. 2 can shed light on the relative role of these two subcomponents unlike the basic decomposition in Eq. 1.

**Fig. 1.**
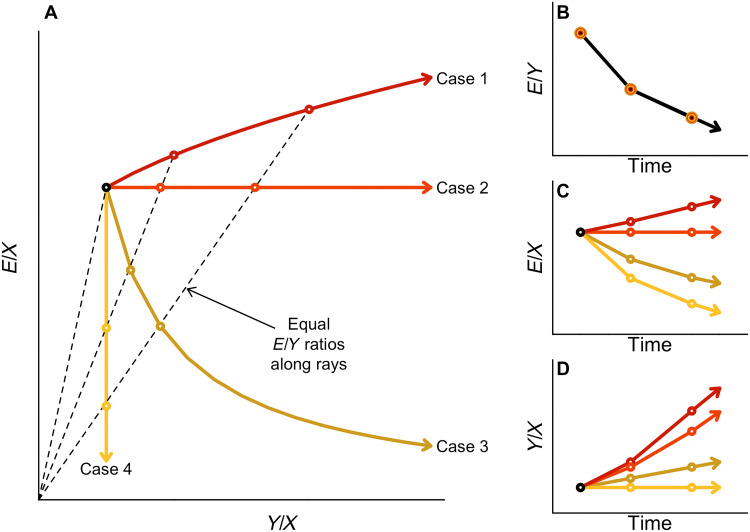
Hypothetical trajectories with identical output emission intensities (E/Y). (**A**) Four hypothetical trajectories with varying degrees of changes in input emission intensity (E/X) and TFP (Y/X) over time. (**B**) Output emission intensities (E/Y) over time for the hypothetical trajectories. These are observationally equivalent. (**C**) Input emission intensities (E/X) over time for the hypothetical trajectories. (**D**) TFP (Y/X) over time for the hypothetical trajectories.

We showcase the insights from this expanded decomposition in Fig. 1, where we depict four hypothetical pathways of a country’s agricultural sector over time. Figure 1A depicts these pathways in a bidimensional graph showing input emission intensity (E/X) against TFP (Y/X). Each pathway describes the evolution of these two indicators over time. By construction, points along the rays of Fig. 1A have equal output emission intensities (E/Y). Thus, these four hypothetical cases exhibit identical declines in output emission intensity over time, as shown in Fig. 1B, and captured by rays with declining angles in Fig. 1A. That is, these four hypothetical cases would appear indistinguishable to observers narrowly focused on E/Y and relying on the decomposition shown in Eq. 1.

The expanded decomposition illustrates how the decline in output emission intensity (E/Y) can take different underlying forms. For instance, case 1 in Fig. 1A achieves the decline in E/Y by rapidly increasing TFP but while also increasing input emission intensity. Case 2 achieves the exact same evolution of E/Y by increasing TFP alone. Case 3 achieves the same decline in E/Y by reducing input emission intensity more than TFP. Last, case 4 achieves the same progress in E/Y by reducing input emission intensity alone without any gains in TFP growth. Figure 1 (C and D) shows the evolution of input emission intensity and TFP, respectively. Focusing on output emission intensity E/Y alone obscures these pathways and thus cannot provide strategic policy guidance as we discuss later.

## RESULTS

### Historical decoupling of production and GHG emissions

We first document the historical decoupling of agricultural production and GHG emissions. We use production and input data from US Department of Agriculture (USDA) Economic Research Service (ERS) (*27*) and GHG emissions from the Food and Agriculture Organization (FAO) (*27*). We provide more details about the data sources in the Supplementary Materials. The GHG data are available since 1961 for only three categories: emissions from crops, emissions from livestock, and emissions from synthetic fertilizers (*28*). This covers only a subset of emissions from agriculture. Starting in 1990, the FAO provides data on four additional categories including drained organic soils, on-farm energy use, savannah fires, and land use change (see fig. S1). In this study, we focus on trends since 1961 only covering three GHG categories. This favors longer time trends that are not affected by discrepancies in land=use–related GHG emissions (*34*). However, we also report findings based on more complete data and recent periods in the Supplementary Materials.

**Fig. 2.**
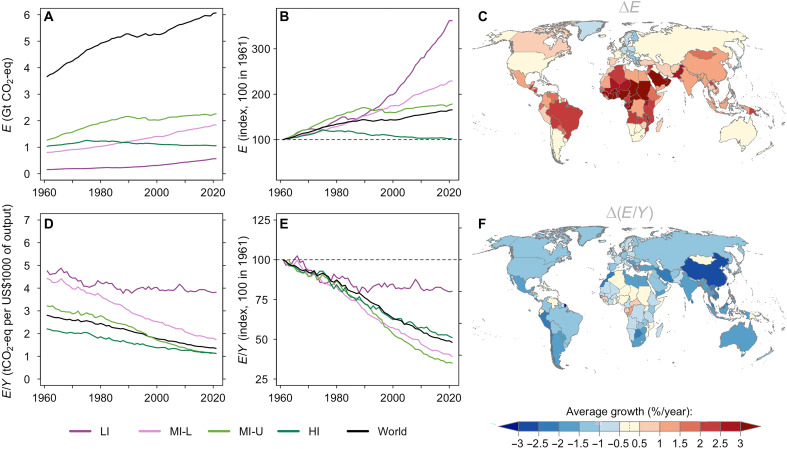
Global GHG emissions and output emission intensities from agriculture (1961 to 2021). (**A**) Evolution of agricultural GHG emission levels (noted *E*) at the global level and by income group. This corresponds to three farm-gate GHG categories with available data over 1961 to 2021: emissions from crops, emissions from livestock, and synthetic fertilizers (see the Supplementary Materials for more details). Income groups correspond to the World Bank classification: low income (LI), lower-middle income (MI-L), upper-middle income (MI-U), and high income (HI) (see the Supplementary Materials for more details). Gt, billion tonnes; eq, equivalent. (**B**) Relative growth of agricultural GHG emissions (relative to a 1961 baseline). (**C**) Map of average country-level agricultural GHG emission growth over the sample period. (**D**) Evolution of agricultural output emission intensity (noted E/Y, where Y is agricultural output), which is the level of GHG emissions per US $1000 worth of output. (**E**) Relative growth in output emission intensity (relative to a 1961 baseline). (**F**) Map of country-level average output emission intensity growth over the sample period.

Global agricultural production has more than tripled since 1961 (+270%), growing at an average pace of about 2.2% year^−1^ (table S2). This growth is somewhat slower in high-income countries (1.3% year^−1^) relative to low- to middle-income countries (2.8 to 3.1% year^−1^) (table S2 and fig. S2). Global GHG emissions from the agricultural sector have also increased over that period but much less so than production (+45%) (Fig. 2A and table S2). In addition, emission growth has been relatively slower in higher-income nations (see Fig. 2, B and C, and table S2). Over 1961 to 2021, while emissions remained stagnant for high-income countries (0.02% year^−1^), they grew at a fast 2.2% year^−1^ in low-income countries. Recall that emission trends since 1961 do not account for all GHG emissions from agriculture (see fig. S1). Figure S3 showcases the trends in GHG growth since 1990 accounting for all seven GHG categories, while fig. S4 maintains the same three original GHG categories since 1990 (see also tables S3 and S4). The emission trends remain qualitatively similar, with emission growth not exceeding the growth of agricultural output.

A salient feature of these trends is that emissions are growing more slowly than output. This is a manifestation of the so-called decoupling that is reflected in reduction in output emission intensity over time across the board (Fig. 2D). This decline in output emission intensity is relatively less pronounced in low-income countries where this ratio has stagnated since the 1990s (Fig. 2E). China exhibits one of the largest declines among large producers with a 2.5% year^−1^ decline over 1961 to 2021 (Fig. 2F and table S2). The declines are even larger when we consider more GHG emission categories since the 1990s especially for Brazil (table S3 and fig. S3F).

### Unpacking the historical decoupling pathways by incorporating inputs

**Fig. 3.**
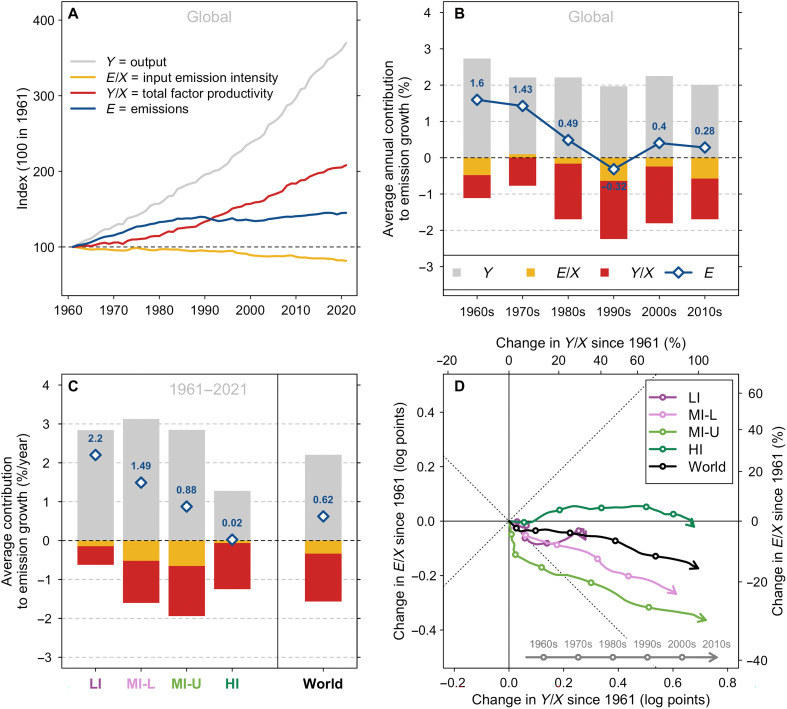
Decomposition of agricultural GHG emission growth. (**A**) Growth of global key indicators (relative to a 1961 baseline) including output (Y), input emission intensity (E/X), TFP (Y/X), and GHG emissions (E). (**B**) Global contributions of the growth of key indicators to GHG emission growth, by decade. (**C**) Average contributions of key indicators to GHG emission growth since 1961, by income level. (**D**) Evolution of input emission intensity and TFP over time, by income level. This is an empirical version in relative terms of the hypothetical trajectories showcased in Fig. 1.

Our proposed decomposition indicates that declines in output emission intensity E/Y can be achieved by either reducing input emission intensity, E/X, or boosting TFP, Y/X. Figure 3A shows indices of the evolution of output (Y), emissions (E), input emission intensity (E/X), and TFP (Y/X). The rise of output (in gray) outstrips the growth of emissions (in blue). During the entire period, E/X declined by just 18.3%, whereas Y/X grew by 108.1% (table S2).

Figure 3B shows the contribution to global emission growth by decade. Global emission growth declined from about 1.6% year^−1^ in the 1960s to about 0.28% year^−1^ in the 2010s. The main positive contributor to emissions is naturally output growth (gray bars). In addition, TFP growth (red bars) has remained the primary driver reducing emissions across decades. Changes in input emission intensity have played a more mixed and limited role over time (orange bars).

TFP growth also remains the main driver of emission reduction across all income levels (Fig. 3C). The relative role of TFP growth in these reductions is largest in high-income countries (1.19% year^−1^) where it has largely offset contributions from output growth (1.27% year^−1^), thus leading to stagnating emissions (0.02% year^−1^) (table S2). Declines in input emission intensity have played a relatively larger role in reducing emissions in lower-middle and upper-middle income countries relative to other income groups (Fig. 3C). We can also appreciate these trends for each income group in figs. S5 to S7.

We also depict changes in input emission intensity against TFP over time by income group in Fig. 3D. This is an empirical analog to the conceptual Fig. 1A but in changes rather than levels. Figure 3D shows that increases in TFP tend to dwarf changes in input emission intensity. The pathway of high-income countries seems dominated by TFP growth with increases in emissions’ growth between the 1960s and the 1990s. However, there is a change in momentum in both high-income and low-income countries toward reductions in input emission intensity since the 2000s. In contrast, the pathways associated with lower-middle and upper-middle income countries remain stable.

When we consider a larger set of emissions since 1990, results remain qualitatively similar (see fig. S8 and table S3). Globally, TFP growth remains the primary factor reducing emissions except for low-income countries (fig. S8, B and C). Much of the emission reduction since the 2000s in low-income countries appears driven by reductions in input emission intensity rather than TFP growth (fig. S8D and table S3). We also show the decomposition for the limited set of GHG emissions since 1990 for comparison purposes (fig. S9 and table S4). The trends appear qualitatively comparable to Fig. 3 and table S2 except for low-income countries. This suggests that there are sizeable changes in input emission intensity associated with the additional GHG categories introduced in 1990.

### Decoupling pathways vary by region and income

**Fig. 4.**
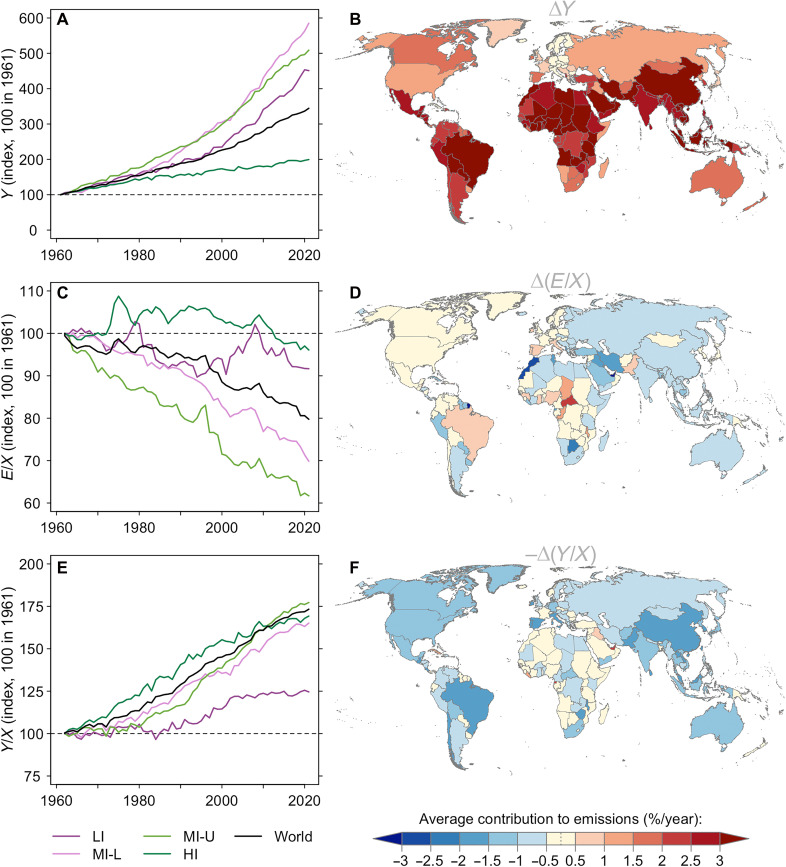
Regional and country-level decomposition of agricultural GHG emission growth. (**A**) Evolution of agricultural output by income group relative to a 1961 baseline. (**B**) Map of average country-level agricultural output growth over 1961 to 2021. (**C**) Evolution of input emission intensity (E/X) by income group relative to a 1961 baseline. (**D**) Map of average country-level input emission intensity change over 1961 to 2021. (**E**) Evolution of TFP (Y/X) by income group relative to a 1961 baseline. (**F**) Map of average country-level contribution of TFP growth to emissions over 1961 to 2021.

Figure 4 shows the evolution of key GHG emission drivers. Figure 4 (A and B) shows that output has grown the least in high-income countries. We also see in Fig. 4 (C and D) that contributions from input emission intensity have been more pronounced in middle-income countries. On the other hand, contributions from TFP growth are relatively larger and affect most countries except low-income countries. These countries, mostly located in Africa, have experienced the slowest TFP growth.

Figure 4 unpacks the improvements in output emission intensity shown in Fig. 2 (D and E). There is no clear relationship between countries exhibiting declines in input emission intensity (Fig. 4D) and those exhibiting faster TFP growth (Fig. 4F). This suggests that nations are undertaking distinct pathways as illustrated in Fig. 1. That is, improvements in output emission intensity shown in Fig. 2F can result from different relative contributions from input emission intensity and TFP growth.

We find these regional patterns remain qualitatively similar when we consider a broader range of GHG emissions since 1990 (fig. S10). The exception is that we see larger improvements in input emission intensity in low-income countries (fig. S10, C and D, versus Fig. 4, C and D). This distinction appears driven by trends related to the additional GHG categories introduced in 1990 rather than the shorter time frame (see fig. S11, C and D).

These pathways vary widely across large producing regions or countries (fig. S12). Emission growth in the United States is small, and this appears primarily driven by TFP growth except for past decade where improvements in input emission intensity were the dominant factor and TFP growth has stagnated. For China, emission growth has declined considerably over time and is now negative, driven by a combination of TFP growth, the main driver, and reductions in input emission intensity. Emission growth in India, in contrast, has not declined; however, TFP remains the primary driver offsetting that growth. Brazil exhibits a different trajectory, with declining emissions that are partly driven by TFP growth. However, input emission intensity has risen when not accounting for changes in land use (not accounted for here but in fig. S13). Emissions in the European Union (EU) have declined, and that is also mostly driven by TFP growth with efforts to reduce input emission intensities in the 1990s and 2010s.

Focusing on a broader set of GHG emissions since 1990 points to comparable patterns for most of these countries and regions (fig. S13). A notable exception is Brazil, for whom emissions appear to decline substantially, driven by reductions in emissions associated with land use change around 2010. That pattern for Brazil is absent when we consider a narrow set of emissions that excludes land use change over the same period (fig. S14). On the contrary, in that case, input emission intensity appears to rise.

We can draw parallels between the theoretical pathways described in Fig. 1 and the empirical pathways of specific countries. Case 1 in Fig. 1 (increases in TFP coupled with emission increases) may be exemplified by the United States in the 1960s and 1970s (fig. S12) when mechanization and other labor-saving technologies led to sustained TFP growth while increasing emission intensity. At the other end of the spectrum, case 4 (pure drop in emission intensity without increases in TFP) could be exemplified by the Former Soviet Union (FSU) between the 1960s and 2010s (fig. S12, right plot) when major changes in input composition occurred (lower animal numbers and area but better seed varieties) without noticeable decreases in TFP but lowering emission intensity.

The other intermediate cases in Fig. 1 roughly correspond to different periods in the EU’s pathway (fig. S12, right plot). For example, case 2 (pure increase in TFP without changes in emission intensity) resembles the 1990s when the EU’s TFP growth dominated any reductions in input emissions’ intensity. Case 3 (reductions in input emission intensity coupled with slower TFP increases) resembles the EU’s pathway in the 2000s. During this period, the EU Common Agricultural Policy decoupled subsidies from production and began requiring compliance with environmental, food safety, and animal welfare standards.

### Underlying trends in input and output mixes

Identifying that TFP growth is the main driver of GHG emission reduction rather than the decrease in input emission intensity does not tell a complete story regarding the underlying changes in inputs, outputs, and technological changes at play. For instance, our findings could be partly driven by trends in changing input or output mixes, or they could be driven by a disproportionate rise in the productivity of certain inputs via processes that are difficult or slow to “decarbonize.” To shed a deeper light on the decoupling pathways identified in Figs. 3 and 4, we explore the trends in various indicators, including specific inputs and outputs, partial productivities, and partial input emission intensities.

We first focus on the evolution of the input mix. Figure S15 (first column) shows that, across all income levels, materials and capital inputs are growing faster than both labor and land. The higher the regional income, the slower the relative growth in the land and labor input. We only observe declines in the labor input in the high-income region and in the upper-middle income region starting in the 2000s. As a result, the input mix and their associated cost shares have become relatively more concentrated in capital and materials over time (table S1).

On the output side, fig. S15 (second column) shows that the growth of crops and livestock has remained similar within each income region, with a slightly faster growth in livestock production in upper-middle income countries and more recently in lower-middle income countries. This may be partially driven by the change in diets toward more animal protein, as income rises in middle-income countries. The changes in the output mix remain relatively more subdued than changes on the input side. This suggests that the underlying drivers of variations in GHG emission growth may be more associated with changes related to input mixes, their productivities, and emission intensities. However, changes in the composition of livestock production with varying levels of emission intensity (growth of poultry and pork relative to beef and sheep) are also likely contributing to these observed patterns.

### Partial decoupling pathways suggest key role for land and labor

In general, changes in the quantities of input used or demanded are related to technological changes that alter the relative return to these inputs. We thus explore the changes in partial input productivities, which are defined as the change in the total output divided by the quantity of an input. A key trend emerging from fig. S15 (third column) is the rising average productivities of land and labor across the board. Labor productivity has grown faster than land productivity in high-income countries but also in upper-middle income countries, especially since the 2000s. In poorer regions, the growth of labor and land productivities is similar with slightly higher land productivity growth. The partial productivities of capital and materials have remained flat in high-income countries but have declined in other regions. This is characteristic of periods of transition of input “deepening” when the share of certain inputs rises disproportionately.

We then explore the evolution of partial input emission intensities, defined as the total emissions divided by individual input quantities. A clear pattern emerges in fig. S15 (fourth column), indicating that partial emission intensities are in decline across inputs but have slightly increased for labor and land input intensities. The strongest evolution is the rise in the emission intensity of labor in high-income countries and in upper-middle income countries since the 2000s. Overall, we find that our observations remain similar when we consider slightly shorter periods and a higher number of GHG categories when describing emissions (figs. S16 and S17).

**Fig. 5.**
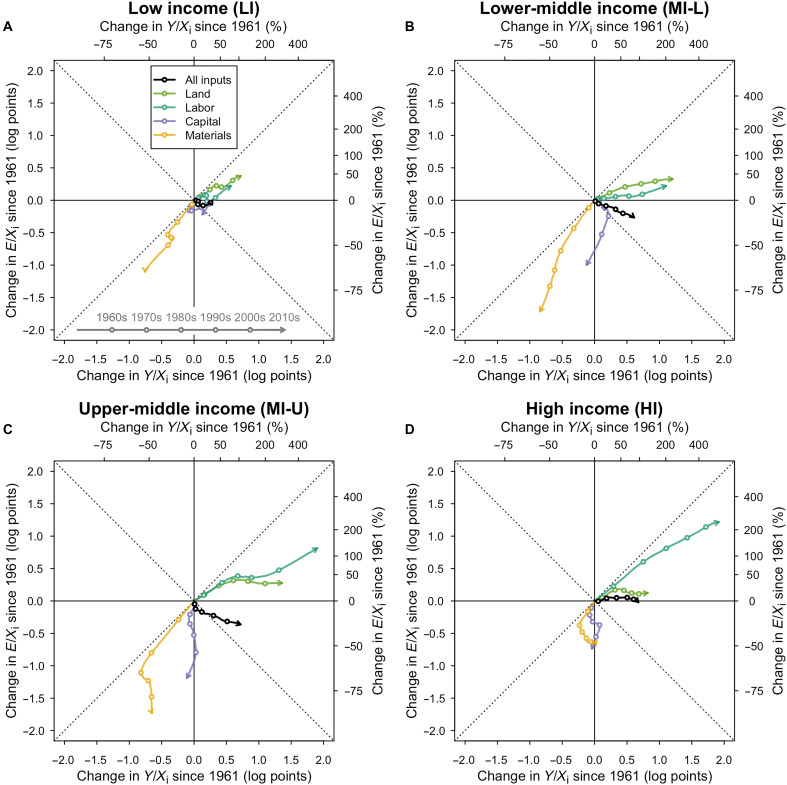
Relative changes of partial input emission intensities and factor productivities by income group. Each panel showcases the evolution of the overall input emission intensities and TFP shown in Fig. 3D in black. We then represent the partial emission intensities ($E/X_{i}$) and partial factor productivities ($Y/X_{i}$) in colored lines. (**A**) LI. (**B**) MI-L. (**C**) MI-U. (**D**) HI.

We explore these technological trends relating specific inputs to output and emissions in Fig. 5 where we visualize partial productivities against partial input emission intensities by income group (third versus fourth columns of fig. S15). That is, we unpack the decoupling pathways of TFP (Y/X) and input emission intensity (E/X), which we highlighted in Fig. 3D and conceptually in Fig. 1. Each panel showcases, in black, the aggregate pathways by income group and, in colors, the pathways for each of the partial input productivities ($Y/X_{i}$) and input emission intensities ($E/X_{i}$). Intuitively, the black aggregate pathway is an average of the colored partial input pathways, weighted by their respective cost shares over time (table S1).

Figure 5 shows some key regularities across income groups. Partial input emission intensities only appear to rise for land and labor, the two inputs for which average productivity is also rising. Input emission intensities for capital and materials appear to decline. We focus more closely on Fig. 5 (C and D), which corresponds to the two income groups with the most different decoupling pathways in Fig. 3D. It appears that the faster rise in the labor emission intensity in the high-income group (Fig. 5D) relative to the upper-middle income group (Fig. 5C) partly explains the small contribution of the E/X channel for reducing emissions of high-income countries. We find somewhat similar trends when we consider more GHG categories (fig. S18), although a look at a restricted number of categories since 1990 suggests that upper-middle income countries are following a very similar pathway to the high-income group, with rapidly rising labor emission intensities (fig. S19).

This pattern is consistent with long-run processes in agricultural development described earlier in the paper (*22*). As economies modernize, industrial inputs such as fertilizers, irrigation, and machinery increasingly substitute traditional factors such as land and labor. This shift tends to lower emission intensity where land-saving innovations dominate but can raise it when mechanization and energy use expand, leading to divergent trajectories across groups of countries. Specifically, we can see how upper-middle income and especially high-income countries exhibit some of the largest increases in labor productivity but also some of the highest increases in the emission intensity of labor.

### Relative changes conceal key level differences

Our analysis thus far has focused on changes in total or partial productivities and how they relate to changes in total or partial input emission intensities. This emphasis on changes may conceal important level differences. We thus explore how partial productivities relate to partial emission intensities in levels. We focus on the two key inputs exhibiting rising productivities, namely, land and labor.

**Fig. 6.**
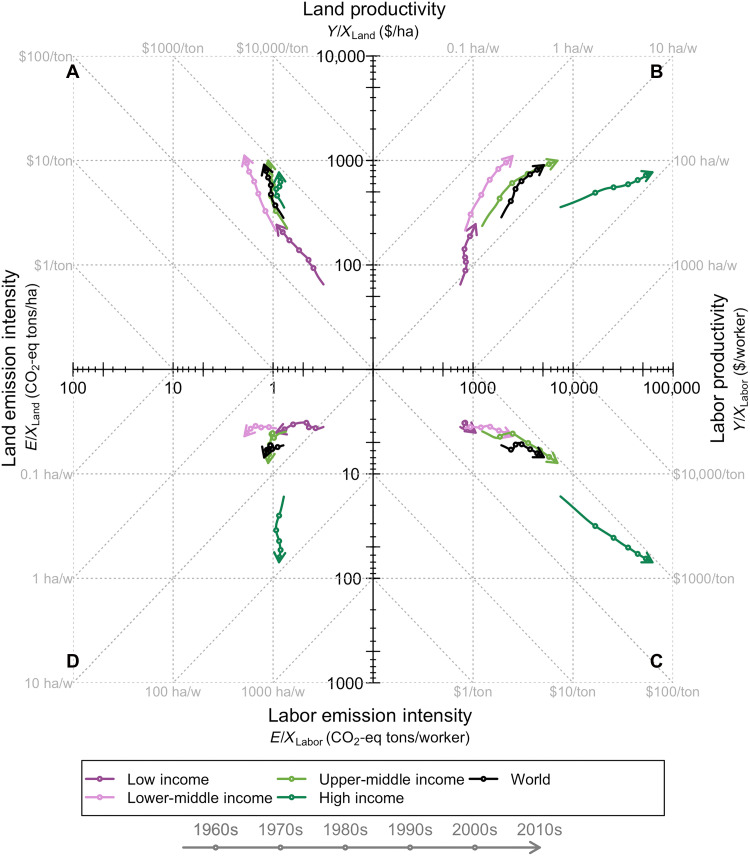
Level changes in partial input emission intensities and factor productivities for land and labor since 1961. Each panel showcases the evolution, in levels, of partial input emission intensities ($E/X_{i}$) and/or partial factor productivities ($Y/X_{i}$) by income group, where the subscript i denotes the input. All axes on log scale. Diagonal dotted lines correspond to fixed ratios in inputs (land and labor) or outputs (marketed output and emissions). (**A**) Land productivity, $Y/X_{\text{Land}}$, versus land emission intensity, $E/X_{\text{Land}}$. (**B**) Land productivity, $Y/X_{\text{Land}}$, versus labor productivity, $Y/X_{\text{Labor}}$. (**C**) Labor emission intensity, $E/X_{\text{Labor}}$, versus labor productivity, $Y/X_{\text{Labor}}$. (**D**) Labor emission intensity, $E/X_{\text{Labor}}$, versus land emission intensity, $E/X_{\text{Land}}$. ha/w, hectares per worker.

Figure 6 has four panels showing how the partial productivities of land and labor relate to the partial emission intensities of these two inputs. Figure 6B shows the historical trajectories of agricultural labor and land productivity across income groups. These trends have been widely documented [e.g., (*35*)]. A key insight is that labor productivity is rising faster in high-income countries (moving rightward more than upward). In addition, middle-income countries have achieved similar land productivity to high-income countries but exhibit noticeably lower labor productivity. However, middle-income countries (especially upper-middle income) appear on a recent trajectory of increasingly faster labor productivity gains, following the path of high-income nations. Low-income nations, on the other hand, are mostly improving land productivity with little to no improvements in labor productivity.

We expand our focus on partial productivities to consider the parallel evolution of input emission intensities. Figure 6 (A and C) indicates how land and labor productivities relate to land and labor emission intensities, respectively. We highlight two key insights. First, gains in land productivity are increasingly being achieved without commensurate increases in land emission intensity (Fig. 6A). This is particularly the case, as country incomes rise. Second, labor productivity is rising alongside commensurate increases in labor emission intensity (Fig. 6C), especially in higher-income countries.

This suggests that technological changes that boost land productivity are more closely linked with reductions in emission intensity and decarbonization than technological changes that boost labor productivity. This makes intuitive sense. Technological changes such as mechanization, while boosting labor productivity ($Y/X_{\text{Labor}}$), do not necessarily alter the biophysical processes that generate GHG as a by-product. In contrast, technologies such as the improved use of fertilizers, which boost land productivity ($Y/X_{\text{Land}}$), would increase output and reduce emission intensity.

Thus, the picture we get from Fig. 3D that high-income countries made little to no reductions in input emission intensity may well be linked to this group’s relatively faster labor productivity growth. However, this relative story hides the fact that lower-income countries now have similar or even higher emission intensities than high-income countries (Fig. 6A).

Last, Fig. 6D showcases how labor and land input emission intensities are changing over time by income groups. This shows that emission intensities in high-income countries are mostly associated with rising labor emission intensity. It is the opposite story for lower-income countries, where increases in land emission intensity are larger than for labor, at least until recently. These findings remain similar when considering more GHG categories and a shorter time frame (figs. S20 and S21).

## DISCUSSION

Our analysis presents several limitations. The emission data from FAO rely on Tier 1 inventory guidelines of the Intergovernmental Panel on Climate Change (IPCC). These emission inventories are rough estimates of actual emissions, so patterns associated with individual countries are less reliable. As a result, our measure of input emission intensity E/X roughly captures changes in input mix over time and not more nuanced improvements in emission factors over time. For instance, it may not capture how the emissions may vary across different dairy herds within the same country.

In addition, while the decomposition we propose sheds light on how the agricultural sector is evolving, it does not provide direct insights into why. Fundamentally, these pathways reflect the evolution of underlying equilibria between growth of Y, X, and E that result from farmer decisions given the available technologies along with market and government incentives. Specifically, farmers choose input bundle X to produce output Y, which inevitably also yields pollution, in this case GHG emissions E.

Our focus on partial productivities and emission intensities sheds some light on the differential role of land and labor. Our analysis suggests that there is more than neutral productivity changes at play. However, we are unable to formally conduct an analysis that partitions GHG emissions into specific inputs or outputs, although GHG emission estimates themselves are based on emission factors that track inputs and outputs. Agriculture is a joint production process converting multiple inputs into multiple outputs, including undesired by-products such as GHG emissions. Multiple combinations of inputs can yield the same output mix, and vice versa. Thus, no input or output is inherently “dirty” or unavoidably tied to GHG emissions. We thus avoid such categorizations to recognize that farmers have the ability, within the current technological possibilities, to reallocate inputs to reduce the level of undesired by-products.

We highlight that country-level decoupling pathways can be influenced in different ways by agri-environmental policies and R&D activities. Some of these factors could have immediate effects (e.g., pricing agricultural GHG emissions or incentivizing carbon sequestration would lead to adjustments with current available technologies) or could take more time to materialize (R&D investments to change the technological frontier). There are indications that gains in labor productivity are not closely linked with reductions in emission intensity. This suggests that land-saving technologies and intensification could point to more promising decarbonization pathways, although other dimensions need to be factored in.

Weighting alternative policy options for decarbonizing agriculture requires rigorous cost-benefit analysis. While the benefits of competing policies are well understood (e.g., the value of higher output and/or lower GHG emissions), estimates of their associated cost remain largely elusive. For instance, although substantial evidence links R&D investment to higher TFP at the national level [e.g., (*35*–*38*)], important gaps remain regarding international spillovers and the effectiveness of directed R&D to reduce emissions. Likewise, global dietary shifts to reduce emissions represent useful aspirational goals, but the cost and effectiveness of policies that achieve such dietary targets must also be evaluated. Future work should focus on building cost-benefit frameworks that help prioritize among competing policy and research strategies to foster a more productive and sustainable global agricultural sector.

## MATERIALS AND METHODS

### Data

#### GHG emissions from agriculture

Data on GHG emissions from agrifood systems come from the FAO FAOSTAT Emissions Totals database. This inventory is computed following Tier 1 methods of the IPCC Guidelines for National Greenhouse Gas Inventories. Emissions correspond to the primary anthropogenic GHGs (methane, carbon dioxide, and nitrous oxide) and fluorinated gasses. The FAO converts all GHG emissions into carbon dioxide equivalents (CO_2_-eq) with global warming potential coefficients from the IPCC Fifth Assessment Report (AR5).

This database provides emission data within the farm gate (excluding energy use) for the 1961–2021 period. Starting in 1990, the database also includes GHG emissions related to land use change. We favor the longer time series (1961 to 2021) for the main analysis in the paper but also report results based on the shorter series in the Supplementary Materials.

Specifically, GHG emission records that start in 1961 include emissions from fertilizer use, crop residues, manure management, manure left on pastures, enteric fermentation, rice cultivation, and savanna fires. Records that start in 1990 include emissions from fires in organic soils and in humid tropical forests, net forest conversion, and emissions from drained organic soils and on-farm energy use. For more information about the categories and their availability, see table 1 and figure 1 in the FAOSTAT Emissions Totals methodology (https://files-faostat.fao.org/production/GT/GT_en.pdf). We show the evolution of these emission categories globally and by country income group in fig. S1. Our focus on farm-gate emissions reflects a commitment to long-term data consistency. We nonetheless provide additional results that incorporate land-use and land-use change emissions in the Supplementary Materials.

Our study analyzes the growth of agricultural emissions as explained by output growth, input emission intensity, and productivity. These are indicators that relate to farms rather than to pre- or postprocessing activities beyond the farm gate. We thus exclude emissions beyond the farm gate in our analysis.

#### Agricultural data

We rely on country-level agricultural production and input data from the USDA ERS International Agricultural Productivity database. This dataset provides country-level estimates of agricultural outputs, inputs, and estimates of TFP. Some of the underlying data in this database originate from FAOSTAT.

TFP growth for country i is measured as the growth of outputs minus the growth of inputs, that is

$$∆{\text{TFP}}_{\mathrm{it}}=\underset{∆Y_{\mathrm{it}}}{\underbrace{\sum_{j=1}^{J}r_{\mathrm{ijt}}∆Y_{\mathrm{ijt}}}}-\underset{∆X_{\mathrm{it}}}{\underbrace{\sum_{k=1}^{K}s_{\mathrm{ikt}}∆X_{\mathrm{ijt}}}}$$

where $\Delta Y_{\mathrm{ijt}}$ and $\Delta X_{\mathrm{ikt}}$ are the growth of output j and input k in year t, respectively, and $r_{\mathrm{jt}}$ and $s_{\mathrm{kt}}$ are their respective revenue and cost shares, which both sum to one. Table S1 shows the cost shares used by USDA ERS. We commonly refer to $\Delta Y_{\mathrm{it}}$ and $\Delta X_{\mathrm{it}}$ as aggregate output and input, respectively. Thus, TFP growth is the growth of output that cannot be explained by the growth of (measured) inputs. To avoid having large swings in prices to affect these revenue and cost shares, the USDA ERS computes average revenue and cost shares over several years. This set up assumes constant returns to scale which is standard.

The USDA ERS relies on outputs that cover 200 commodities reported by FAOSTAT including 162 crops, 30 animal, and 8 aquaculture outputs. In our analysis, we exclude aquaculture outputs because the FAOSTAT Emissions Totals domain data we use do not include the associated GHG emissions. These commodities are aggregated through constant average (2014 to 2016) world prices in purchasing power parity (PPP) dollars. The PPP prices account for differences in purchasing power across different countries in the world. These common world prices reduce the output quantities substantially to a Paasche quantity index. While alternative outcome metrics—such as caloric or protein content—are relevant for assessing food security and sustainability, our approach here seeks to maintain consistency with economic theory and established productivity accounting frameworks.

The input quantity index is an aggregate of land, labor, capital, and material inputs. The aggregation of single input quantities is made through cost shares that are estimated by different sources, vary by region and by decade. The land input is a weighted average of pasture, irrigated, and nonirrigated cropland areas to account for different agricultural production potential. Land areas are expressed in a common denominator in rainfed crop equivalents. The cropland weights represent a coarse classification of differences in land production potential, as they only vary by subcontinent and do not vary over time.

The labor input is the headcount of economically active adult labor force primarily used in agriculture. The USDA ERS relies on data from ILOSTAT, which is the statistical service of the International Labour Organization (ILO) of the United Nations. Neither workforce quality nor the possibility of off-farm income is accounted for in this input.

The capital input is a measure of changes in net capital stock and also originates from FAOSTAT. Intermediate inputs are composed of a crop and an animal aggregate. The crop aggregate follows the changes in quantities of inorganic and organic fertilizers. The animal aggregate follows the trends in animal feed. Animal feed from all sources (except hay and fodder) is measured in terms of total energy content of feeds.

#### Country income classification

At various points in the paper, we present results by broad income categories. For this, we rely on the World Bank Group country classification by income level in 2020 (fig. S2). The World Bank country grouping classifies countries into four categories: low income, lower-middle income, upper-middle income, and high income countries. This classification is based on the previous calendar year Gross National Income per capita in purchasing power parities.

In a few figures in the Supplementary Materials, we also create some regional country groups to provide more regional perspectives. We include some figures for the EU and for the FSU. Our EU grouping corresponds to the European Member States in 2021. Our FSU grouping corresponds to the countries belonging to the FSU. Our intention was to present regional patterns with relatively long time series rather than representing groupings with political cohesiveness or unity.

### Methods

Our methodology is simply based on the identity

$$E_{\mathrm{it}}=Y_{\mathrm{it}}\times \frac{E_{\mathrm{it}}}{X_{\mathrm{it}}}\times \frac{X_{\mathrm{it}}}{Y_{\mathrm{it}}}$$

where $E_{\mathrm{it}}$ are GHG emissions in county i and year t, Y is aggregate output, E/X is input emission intensity, and X/Y is the reciprocal of TFP. Taking logs and first differences, we obtain

$$\text{log}\left(\frac{E_{\mathrm{it}}}{E_{\mathrm{it}-1}}\right)=\text{log}\left(\frac{Y_{\mathrm{it}}}{Y_{\mathrm{it}-1}}\right)+\text{log}\left(\frac{E_{\mathrm{it}}/X_{\mathrm{it}}}{E_{\mathrm{it}-1}/X_{\mathrm{it}-1}}\right)-\text{log}\left(\frac{Y_{\mathrm{it}}/X_{\mathrm{it}}}{Y_{\mathrm{it}-1}/X_{\mathrm{it}-1}}\right)$$

which approximates as percentage growth rates as (see Eq. 2)

$$∆E_{\mathrm{it}}\approx ∆Y_{\mathrm{it}}+∆\left(\frac{X_{\mathrm{it}}}{E_{\mathrm{it}}}\right)-∆\left(\frac{Y_{\mathrm{it}}}{X_{\mathrm{it}}}\right)$$

The key point is that decoupling of emission growth $\Delta E_{\mathrm{it}}$ and output growth $\Delta Y_{\mathrm{it}}$ can occur via decreases in input emission intensity $\left(X_{\mathrm{it}}/E_{\mathrm{it}}\right)$ or growth in TFP $\left(Y_{\mathrm{it}}/X_{\mathrm{it}}\right)$ . Our study explores, for each country, region, and globally, the relative contribution of these different components to highlight the existence of various emission intensity-TFP pathways highlighted in Fig. 1.

## Supplementary Material

20260116-1

## Notes

**Correction (27 March 2026):** In the original version of the text, an attribution was omitted for Eq. 2, which closely follows the formula presented in (*25*). The reference is now cited in the text before Eq. 2. The PDF and online versions of the article have been corrected.
