# Supplementary material for: Unpacking the growth of global agricultural greenhouse gas emissions
Source: Sci Adv. 2026 Jan 16;12(3):eaeb8653. doi: 10.1126/sciadv.aeb8653 (PMC12810634; doi:10.1126/sciadv.aeb8653)
Supplement: Supplementary file 2 — Figs. S1 to S21 Tables S1 to S4 [file sciadv.aeb8653_sm.pdf]

Science Advances
AAAS

Supplementary Materials for

Unpacking the growth of global agricultural greenhouse gas emissions

Ariel Ortiz-Bobea and Simone Pieralli

Corresponding author: Ariel Ortiz-Bobea, ao332@cornell.edu

Sci. Adv. **12**, eaeb8653 (2026)
DOI: 10.1126/sciadv.aeb8653

This PDF file includes:

Figs. S1 to S21
Tables S1 to S4

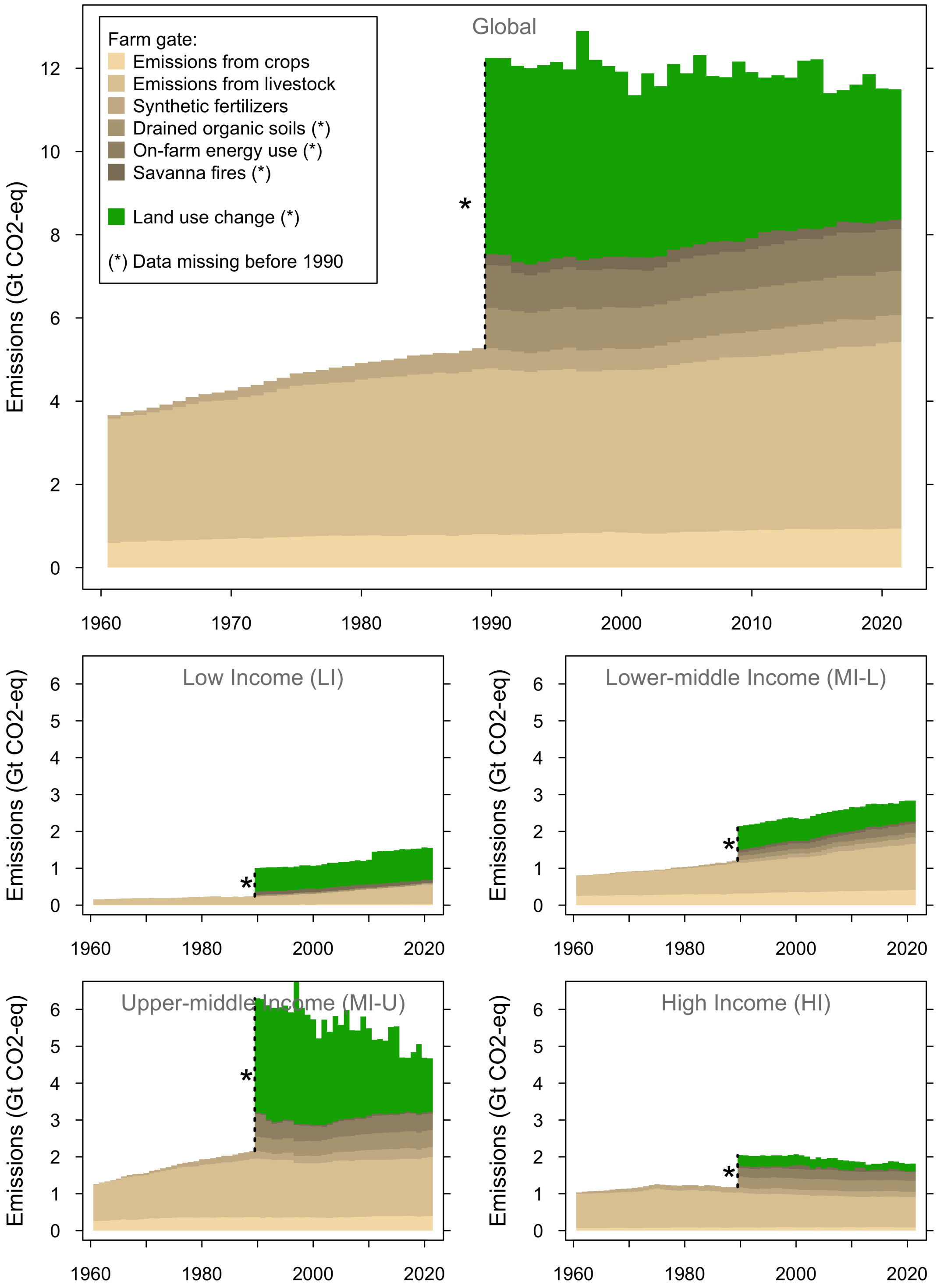

Figure S1: Greenhouse gas (GHG) emissions from agriculture since 1961, globally and by income group.

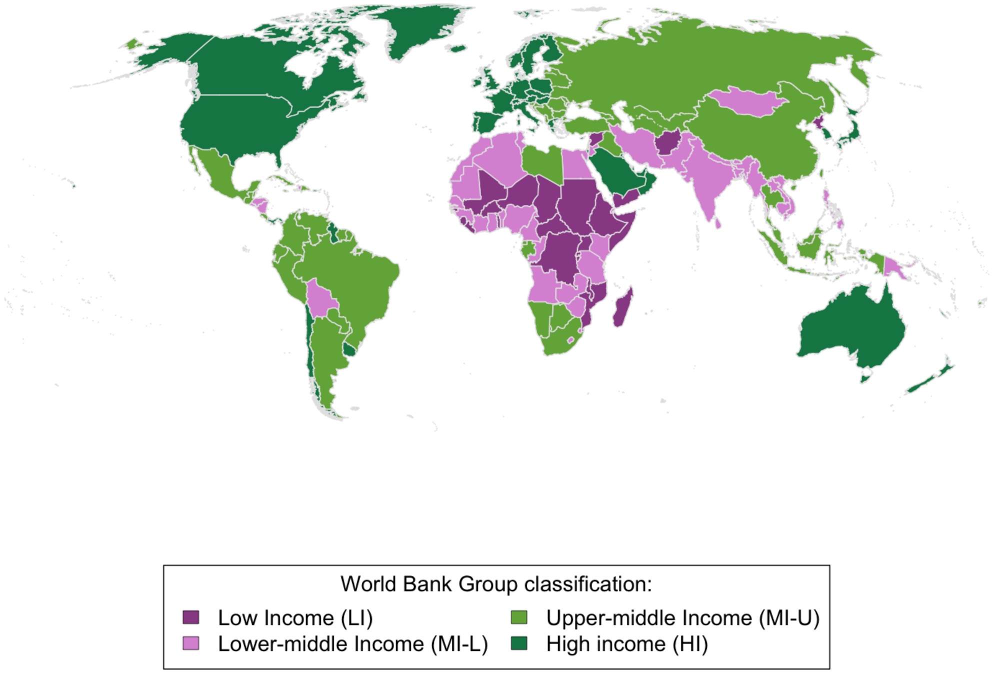

Figure S2: World Bank Group country classification by income level (2020).

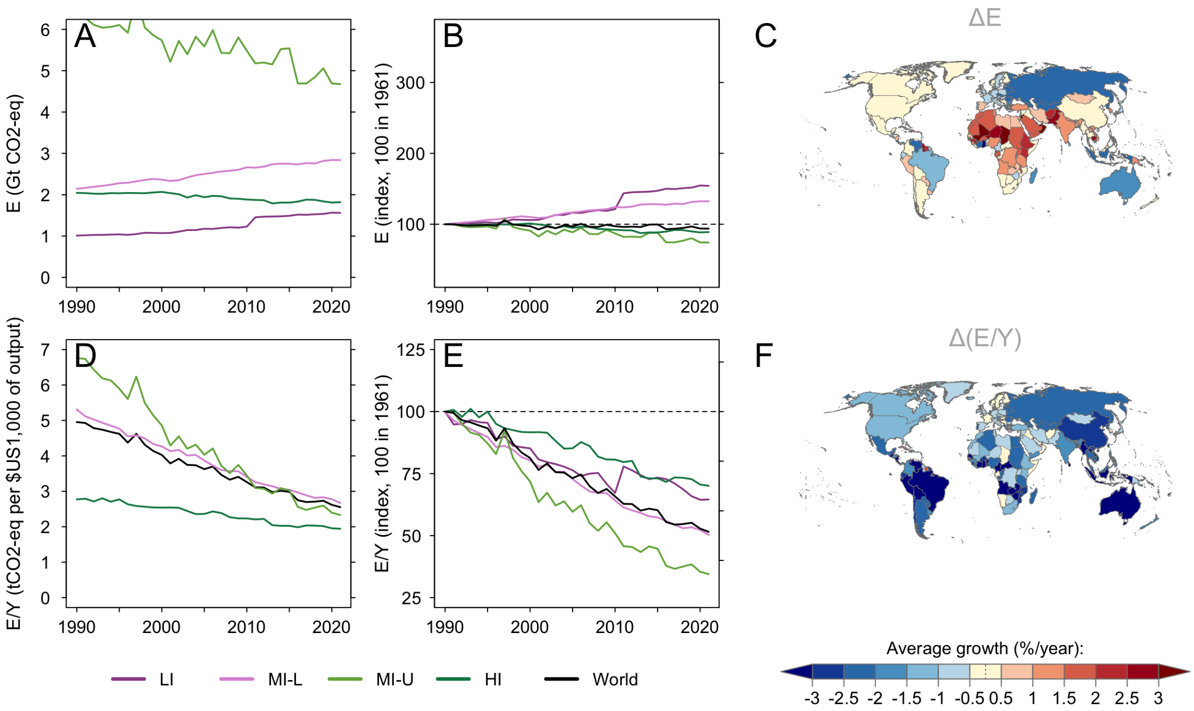

Figure S3: Global greenhouse gas (GHG) emissions and output emission intensities since 1990 (7 GHG categories).

(**A**) Evolution of agricultural GHG emission levels (noted E) at the global level and by income group. This corresponds to all 7 GHG categories with available data over 1990-2021: emissions from crops, emissions from livestock and synthetic fertilizers (available since 1961) as well as drained organic soils, on-farm energy, savannah fires and land use change (available since 1990). (**B**) Relative growth of agricultural GHG emissions (relative to a 1990 baseline). (**C**) Map of average country-level agricultural GHG emission growth over the sample period. (**D**) Evolution of agricultural output emission intensity (noted E/Y, where Y is agricultural output) which is the level of GHG emissions per $US 1,000 worth of output. (**E**) Relative growth in output emission intensity (relative to a 1990 baseline). (**F**) Map of country-level average output emission intensity growth over the sample period.

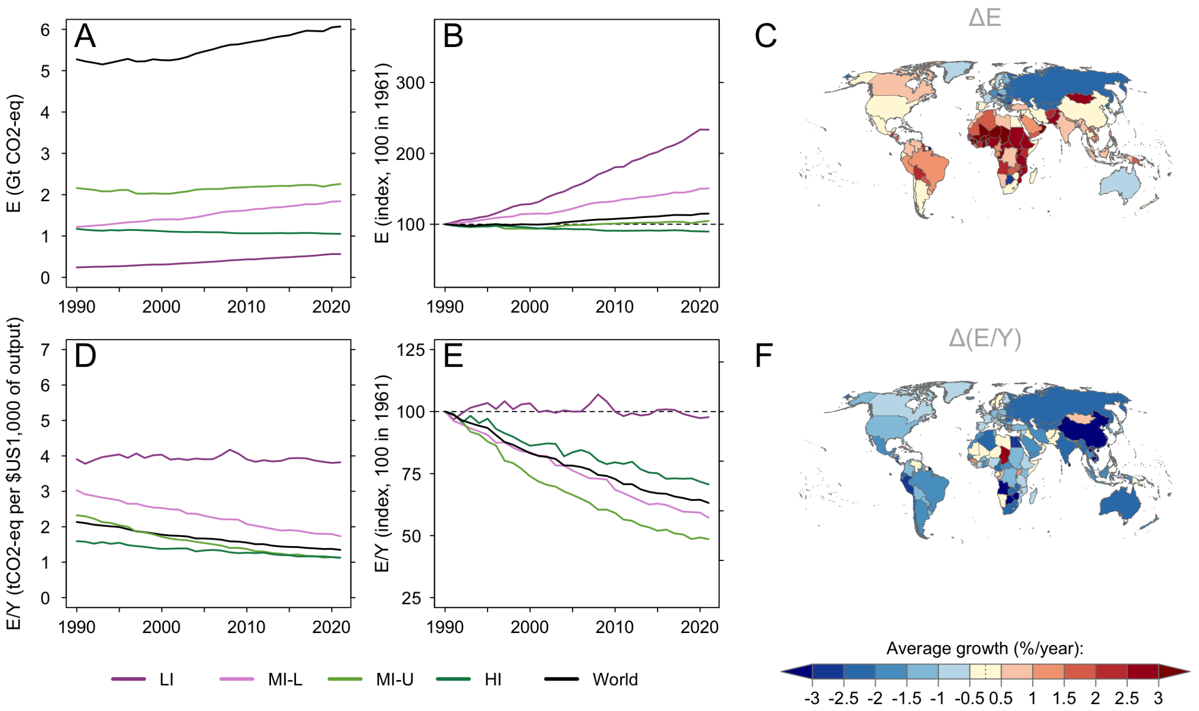

Figure S4: Global greenhouse gas (GHG) emissions and output emission intensities since 1990 (3 GHG categories).

This figure is similar to Fig. S3 but represents only 3 GHG categories for which there is data prior to 1990. (**A**) Evolution of agricultural GHG emission levels (noted E) at the global level and by income group. This corresponds to only the 3 GHG categories with available data since 1961: emissions from crops, emissions from livestock and synthetic fertilizers. (**B**) Relative growth of agricultural GHG emissions (relative to a 1990 baseline). (**C**) Map of average country-level agricultural GHG emission growth over the sample period. (**D**) Evolution of agricultural output emission intensity (noted E/Y, where Y is agricultural output) which is the level of GHG emissions per $US 1,000 worth of output. (**E**) Relative growth in output emission intensity (relative to a 1990 baseline). (**F**) Map of country-level average output emission intensity growth over the sample period.

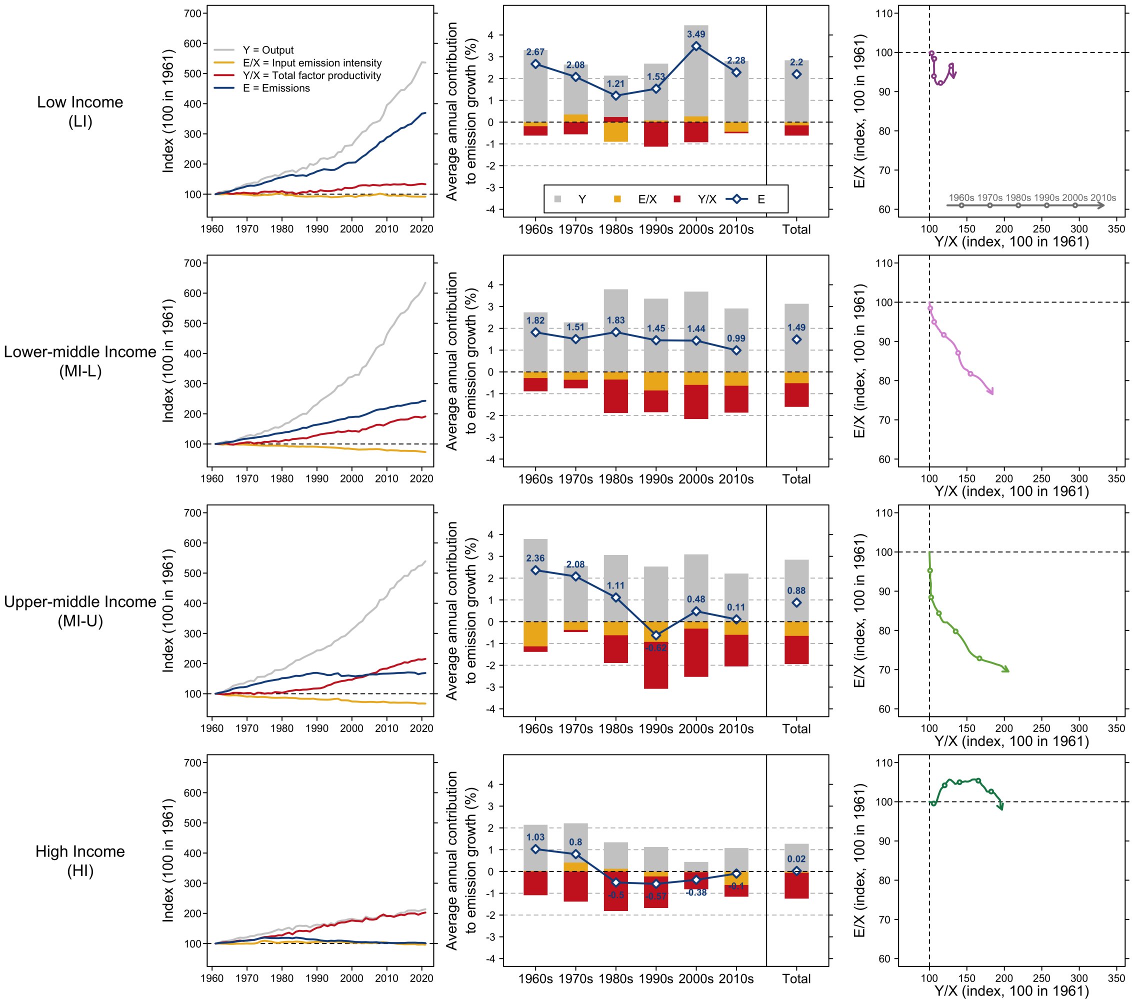

Figure S5: Decomposition of greenhouse gas (GHG) emission growth by income group since 1961 (3 GHG categories).

The GHG emissions here represent only the 3 categories for which there is data starting in 1961 (see Fig. S1). Each row of panels corresponds to a specific income group region. The first column of panels is a regional analogue of Fig. 3A in the main text and depicts the growth of regional key indicators (relative to a 1961 baseline) including output (Y), input emission intensity (E/X), total factor productivity (Y/X) and GHG emissions (E). The second column of panels is a regional analogue of Fig. 3B in the main text and depicts the contributions of the growth of key indicators to GHG emission growth, by decade. The third column of panels is a regional analogue of Fig. 3D in the main text and depicts the evolution of input emission intensity and total factor productivity over time.

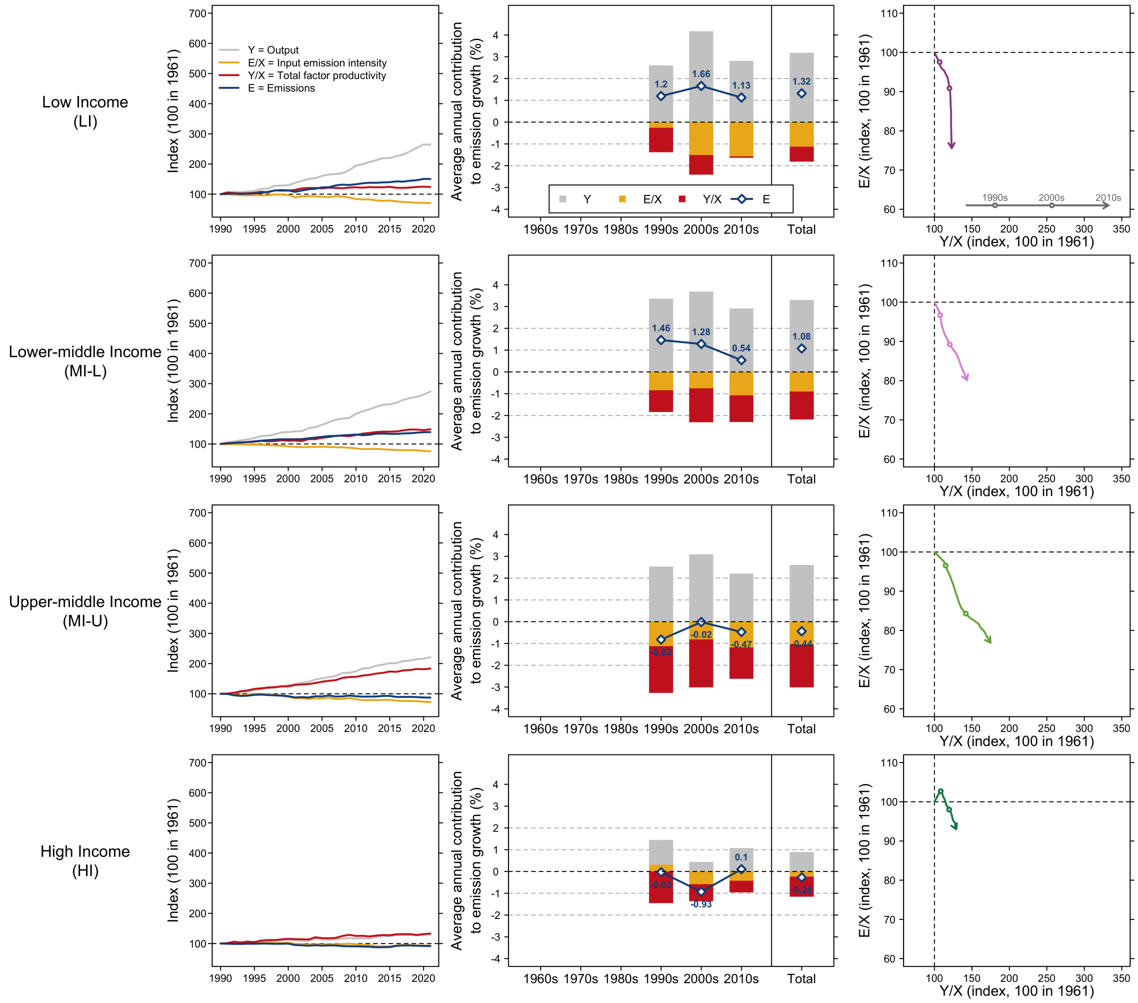

Figure S6: Decomposition of greenhouse gas (GHG) emission growth by income group since 1990 (7 GHG categories).

This figure is analogous to Fig. 3 in the main paper. The GHG emissions here represent the 7 categories for which there is data starting in 1990 (see Fig. S1). Each row of panels corresponds to an income group region. The first column of panels is a regional analogue of Fig. 3A in the main text and depicts the growth of key indicators (relative to a 1990 baseline) including output (Y), input emission intensity (E/X), total factor productivity (Y/X) and GHG emissions (E). The second column of panels is a regional analogue of Fig. 3B in the main text and depicts the contributions of the growth of key indicators to GHG emission growth, by decade. The third column of panels is a regional analogue of Fig. 3D in the main text and depicts the evolution of input emission intensity and total factor productivity over time.

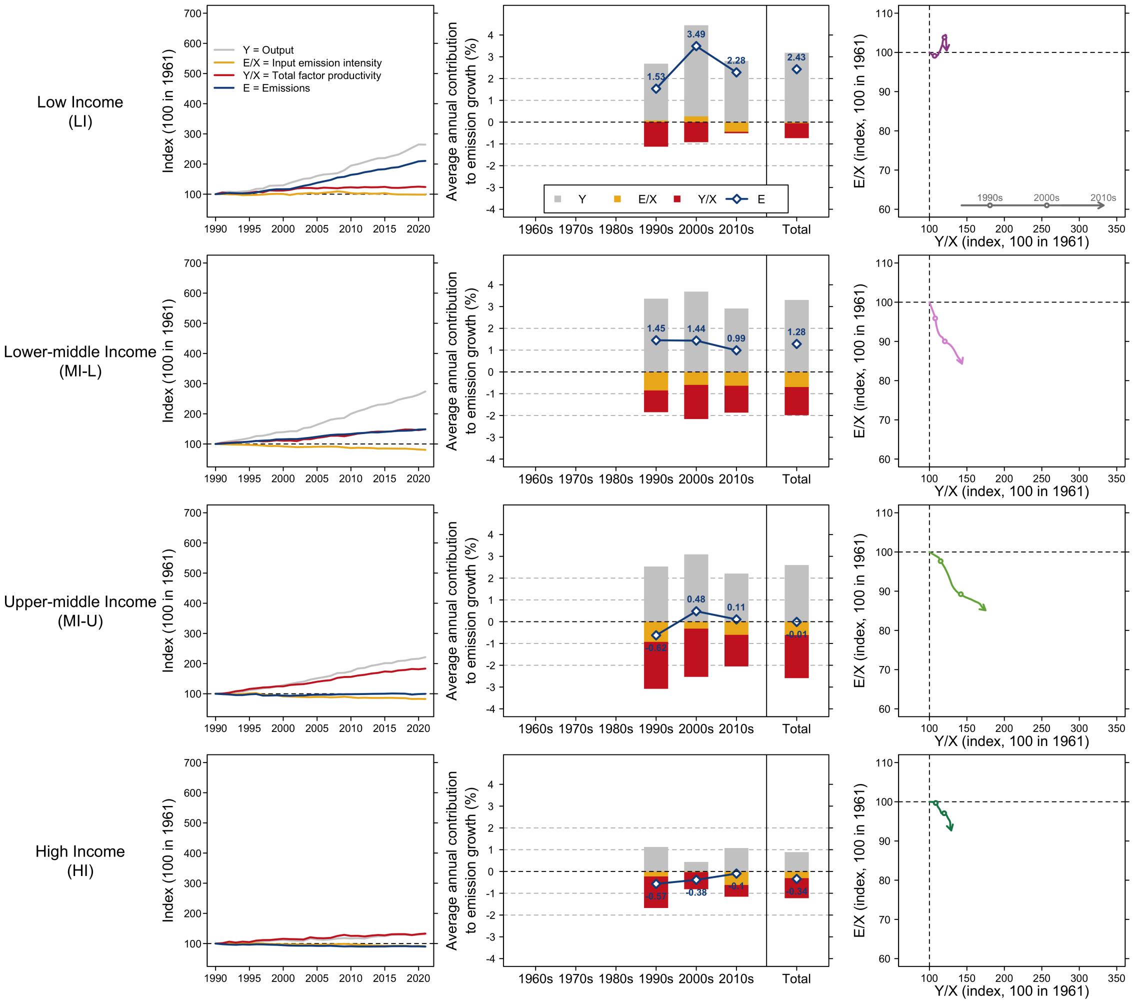

Figure S7: Decomposition of greenhouse gas (GHG) emission growth by income group since 1990 (3 GHG categories).

This figure is analogous to Fig. S6 but relies on 3 GHG categories rather than 7. The GHG emissions here only represent the 3 categories for which there is data starting in 1961 (see Fig. S1). Each row of panels corresponds to an income group region. The first column of panels is a regional analogue of Fig. 3A in the main text and depicts the growth of key indicators (relative to a 1990 baseline) including output (Y), input emission intensity (E/X), total factor productivity (Y/X) and GHG emissions (E). The second column of panels is a regional analogue of Fig. 3B in the main text and depicts the contributions of the growth of key indicators to GHG emission growth, by decade. The third column of panels is a regional analogue of Fig. 3D in the main text and depicts the evolution of input emission intensity and total factor productivity over time.

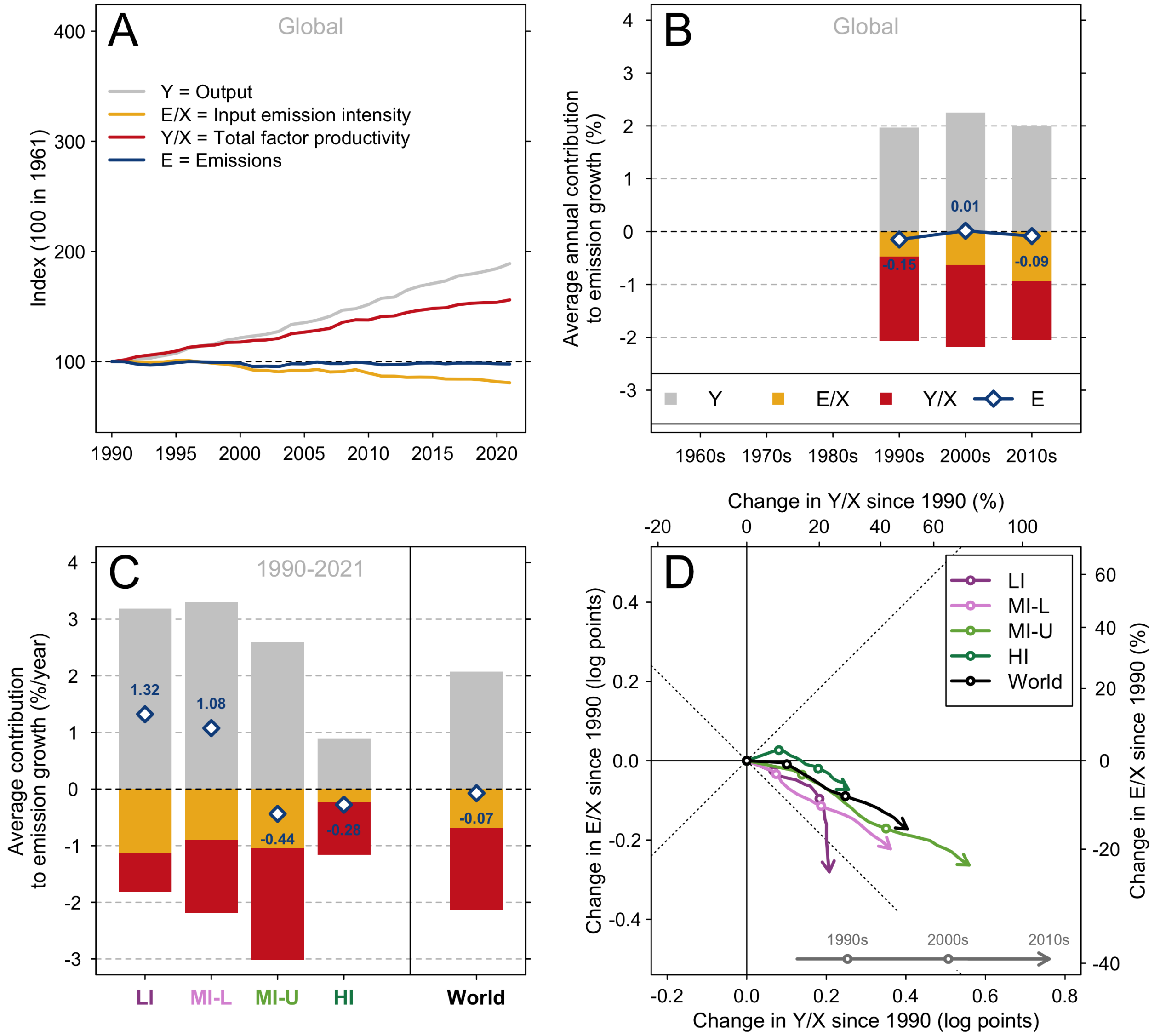

Figure S8: Decomposition of greenhouse gas (GHG) emission growth since 1990 (7 GHG categories).

This figure is analogous to Fig. 3 in the main text. The GHG emissions here represent the 7 GHG categories for which there is data starting in 1990 (see Fig. S1). (**A**) Growth of global key indicators (relative to a 1961 baseline) including output (Y), input emission intensity (E/X), total factor productivity (Y/X) and GHG emissions (E). (**B**) Global contributions of the growth of key indicators to GHG emission growth, by decade. (**C**) Average contributions of key indicators to GHG emission growth since 1961, by income level. (**D**) Evolution of input emission intensity and total factor productivity over time, by income level. This is an empirical version in relative terms of the hypothetical trajectories showcased in Fig. 1. Note that the vertical and horizontal axes have different scales.

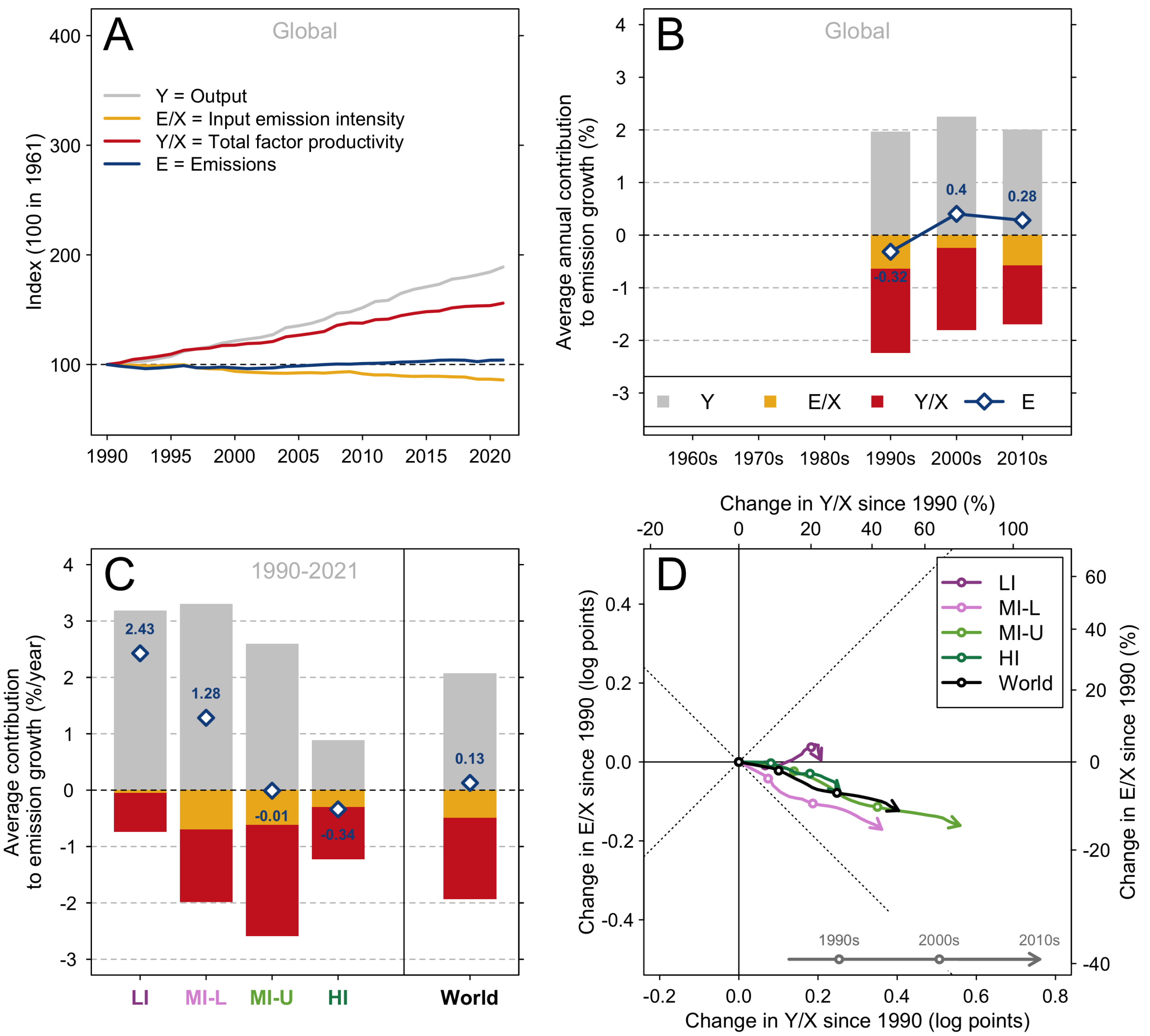

Figure S9: Decomposition of greenhouse gas (GHG) emission growth since 1990 (3 GHG categories).

This figure is analogous to Fig. S8 but relies on only 3 GHG categories rather than 7. The GHG emissions here represent only the 3 GHG categories for which there is data starting in 1961 (see Fig. S1). (**A**) Growth of global key indicators (relative to a 1990 baseline) including output (Y), input emission intensity (E/X), total factor productivity (Y/X) and GHG emissions (E). (**B**) Global contributions of the growth of key indicators to GHG emission growth, by decade. (**C**) Average contributions of key indicators to GHG emission growth since 1990, by income level. (**D**) Evolution of input emission intensity and total factor productivity over time, by income level. This is an empirical version in relative terms of the hypothetical trajectories showcased in Fig. 1. Note that the vertical and horizontal axes have different scales.

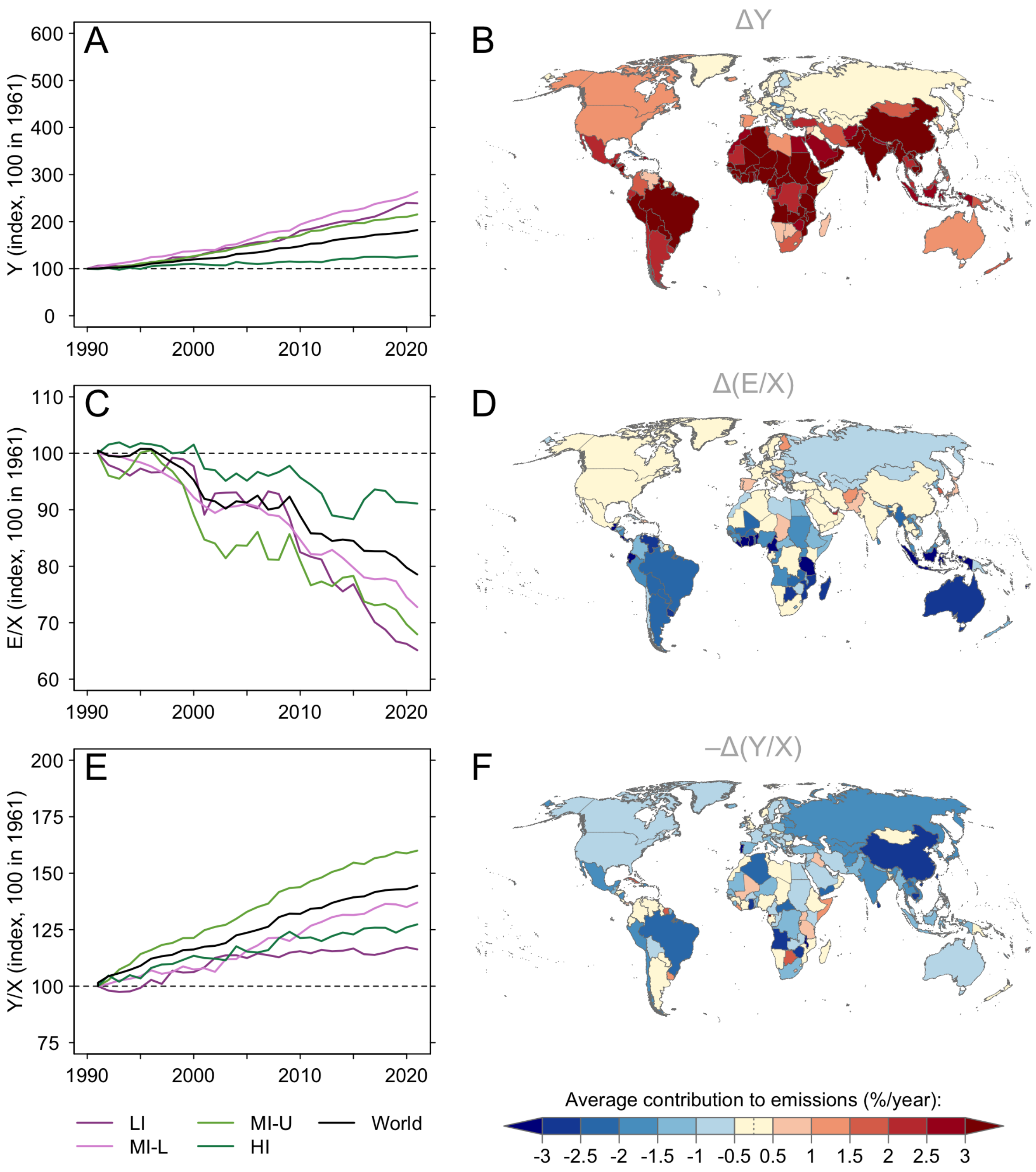

Figure S10: Regional and country-level decomposition of greenhouse gas (GHG) emission growth since 1990 (7 GHG categories).

This figure is analogous to Fig. 4 in the main text. The GHG emissions here represent the 7 GHG categories for which there is data starting in 1990 (see Fig. S1). (**A**) Evolution of agricultural output by income group relative to a 1990 baseline. (**B**) Map of average country-level agricultural output growth over 1990-2021. (**C**) Evolution of input emission intensity (E/X) by income group relative to a 1990 baseline. (**D**) Map of average country-level input emission intensity change over 1990-2021. (**E**) Evolution of total factor productivity (Y/X) by income group relative to a 1990 baseline. (**F**) Map of average country-level contribution of total factor productivity growth to emissions over 1990-2021.

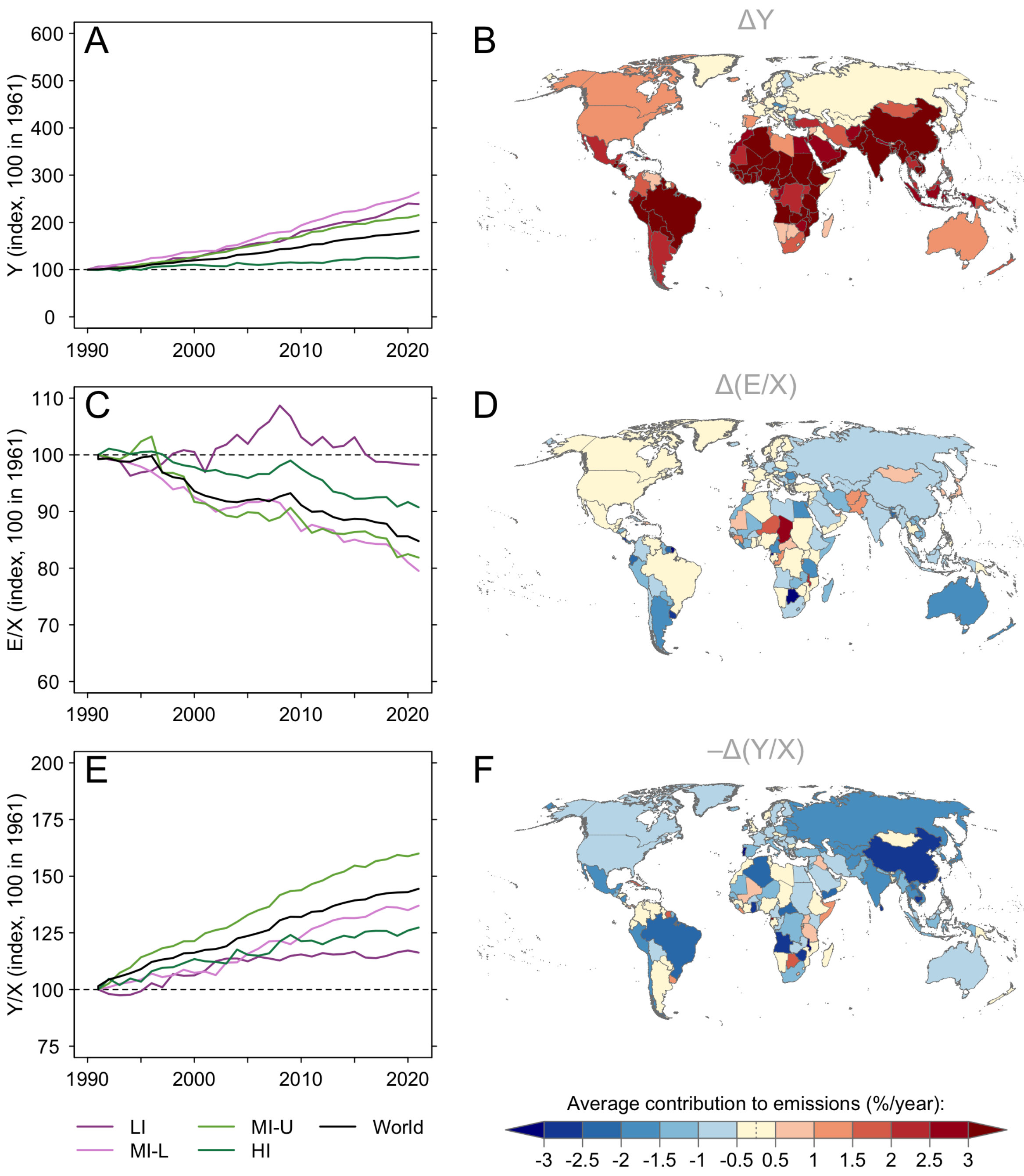

Figure S11: Regional and country-level decomposition of greenhouse gas (GHG) emission growth since 1990 (3 GHG categories).

This figure is analogous to Fig. S10 but relies on 3 GHG categories rather than 7. The GHG emissions here represent only the 3 GHG categories for which there is data starting in 1961 (see Fig. S1). (**A**) Evolution of agricultural output by income group relative to a 1990 baseline. (**B**) Map of average country-level agricultural output growth over 1990-2021. (**C**) Evolution of input emission intensity (E/X) by income group relative to a 1990 baseline. (**D**) Map of average country-level input emission intensity change over 1990-2021. (**E**) Evolution of total factor productivity (Y/X) by income group relative to a 1990 baseline. (**F**) Map of average country-level contribution of total factor productivity growth to emissions over 1990-2021.

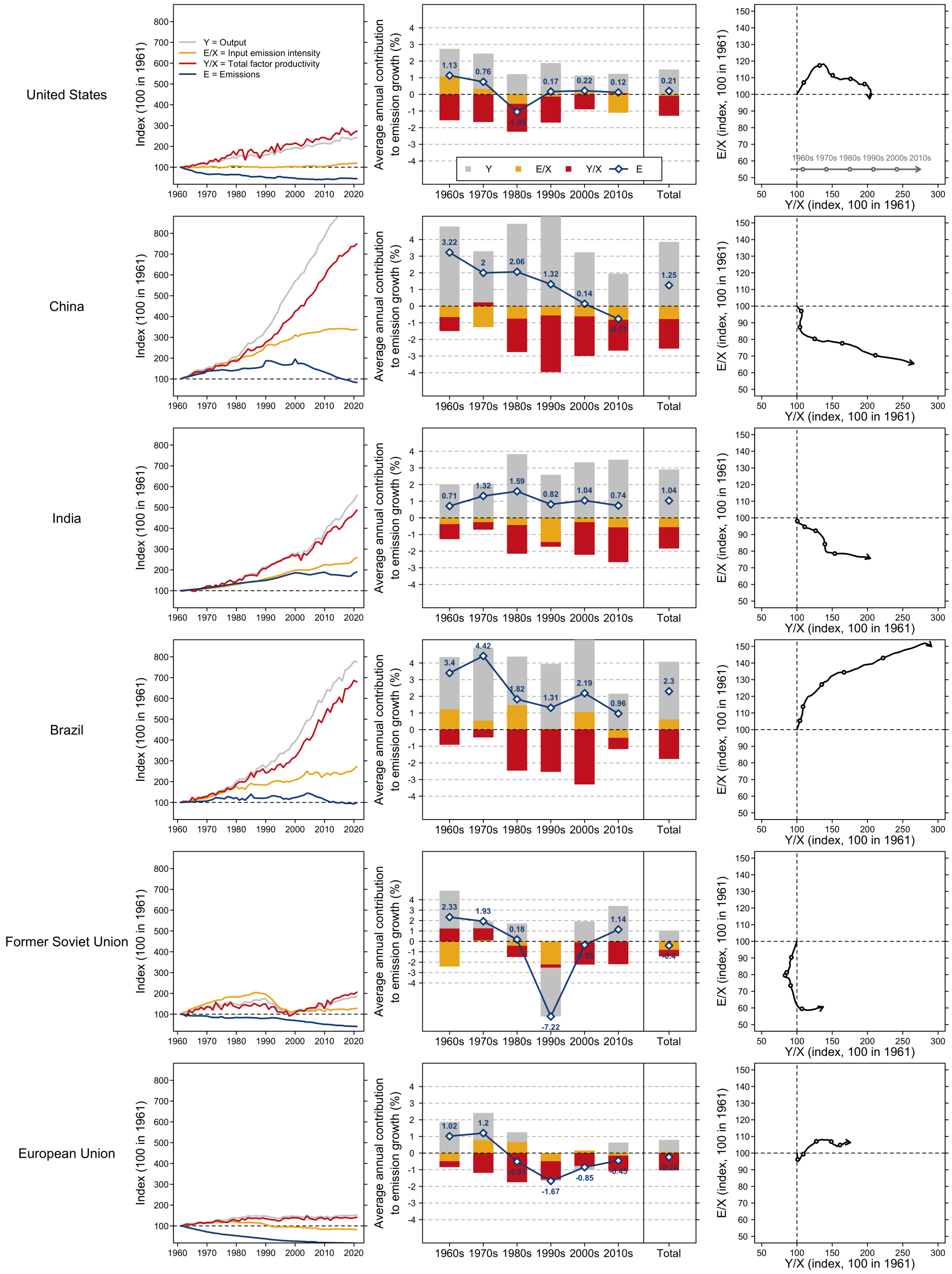

Figure S12: Decomposition of greenhouse gas (GHG) emission growth for select countries since 1961 (3 GHG categories).

This figure is analogous to Fig. 3 in the main paper. The GHG emissions here represent only the 3 categories for which there is data starting in 1961 (see Fig. S1). Each row of panels corresponds to a select country. The first column of panels is a country-level analogue of Fig. 3A in the main text and depicts the growth of key indicators (relative to a 1961 baseline) including output (Y), input emission intensity (E/X), total factor productivity (Y/X) and GHG emissions (E). The second column of panels is a country-level analogue of Fig. 3B in the main text and depicts the contributions of the growth of key indicators to GHG emission growth, by decade. The third column of panels is a country-level analogue of Fig. 3D in the main text and depicts the evolution of input emission intensity and total factor productivity over time.

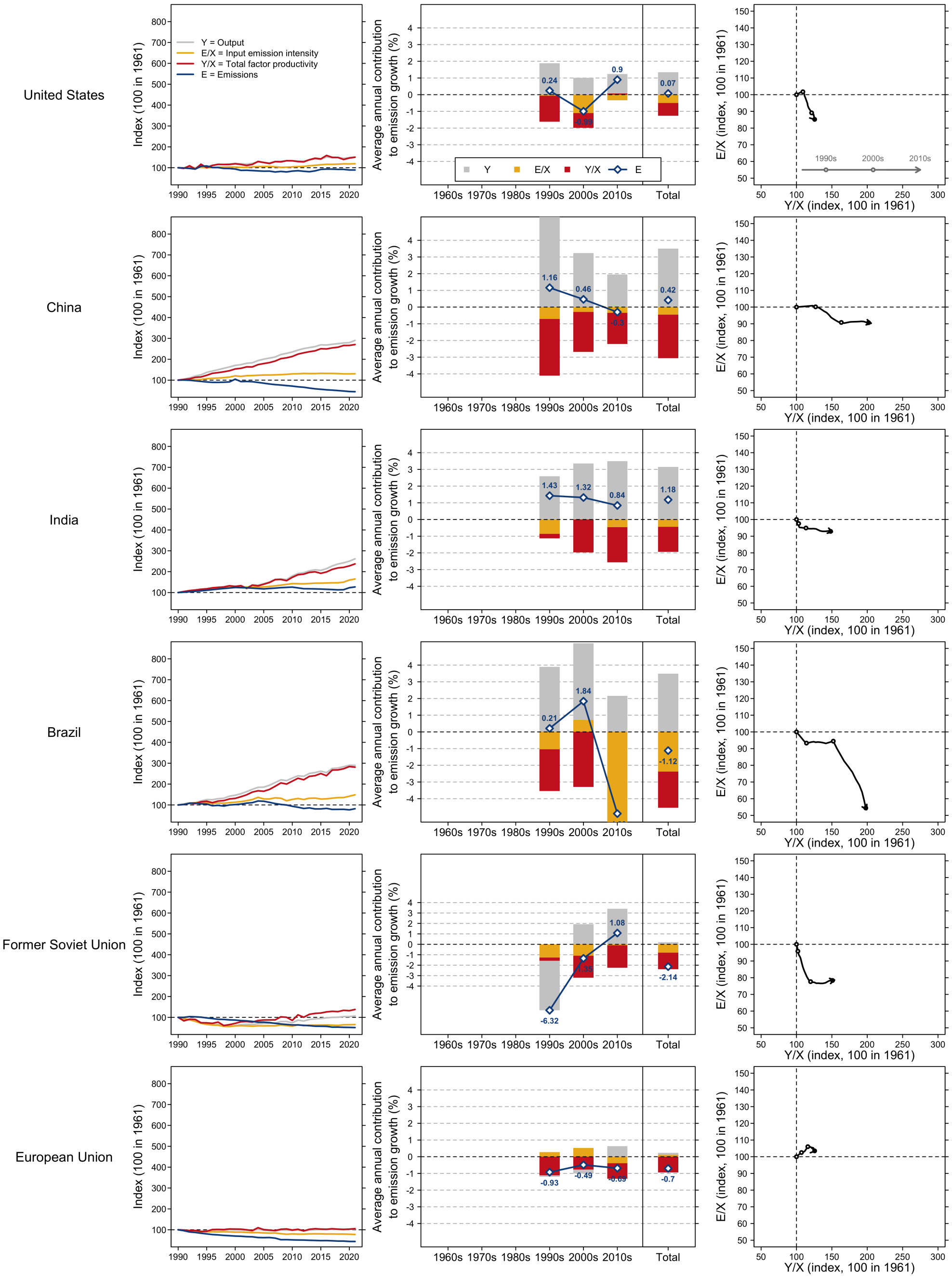

Figure S13: Decomposition of greenhouse gas (GHG) emission growth for select countries since 1990 (7 GHG categories).

This figure is analogous to Fig. 3 in the main paper. The GHG emissions here represent the 7 categories for which there is data starting in 1990 (see Fig. S1). Each row of panels corresponds to a select country or geographical region. The first column of panels is a country-level analogue of Fig. 3A in the main text and depicts the growth of key indicators (relative to a 1990 baseline) including output (Y), input emission intensity (E/X), total factor productivity (Y/X) and GHG emissions (E). The second column of panels is a country-level analogue of Fig. 3B in the main text and depicts the contributions of the growth of key indicators to GHG emission growth, by decade. The third column of panels is a country-level analogue of Fig. 3D in the main text and depicts the evolution of input emission intensity and total factor productivity over time.

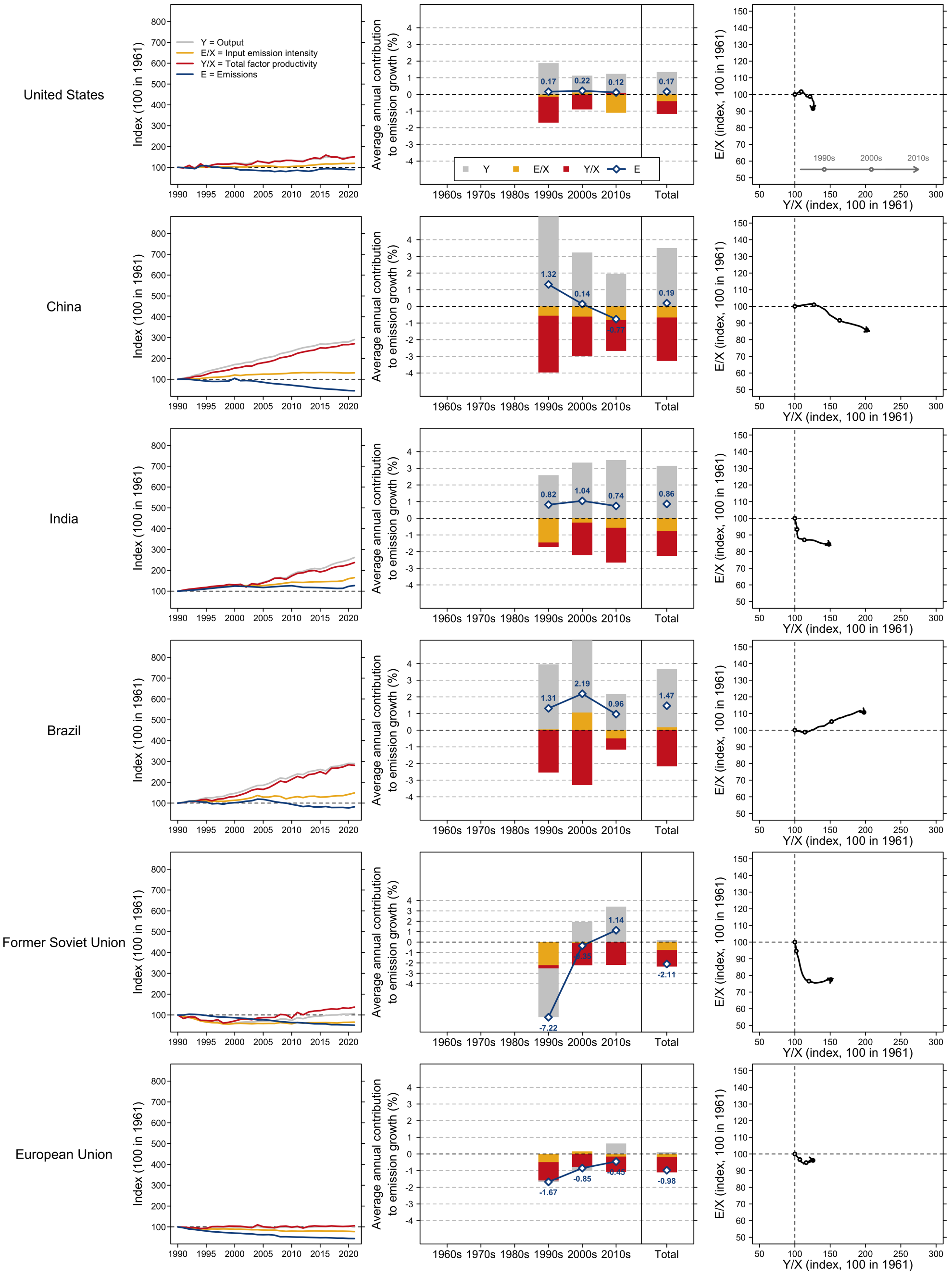

Figure S14: Decomposition of greenhouse gas (GHG) emission growth for select countries since 1990 (3 GHG categories).

This figure is analogous to Fig. S8 but relies on 3 GHG categories rather than 7. The GHG emissions here only represent the 3 categories for which there is data starting in 1961 (see Fig. S1). Each row of panels corresponds to a select country or geographical region. The first column of panels is a country-level analogue of Fig. 3A in the main text and depicts the growth of key indicators (relative to a 1990 baseline) including output (Y), input emission intensity (E/X), total factor productivity (Y/X) and GHG emissions (E). The second column of panels is a country-level analogue of Fig. 3B in the main text and depicts the contributions of the growth of key indicators to GHG emission growth, by decade. The third column of panels is a country-level analogue of Fig. 3D in the main text and depicts the evolution of input emission intensity and total factor productivity over time.

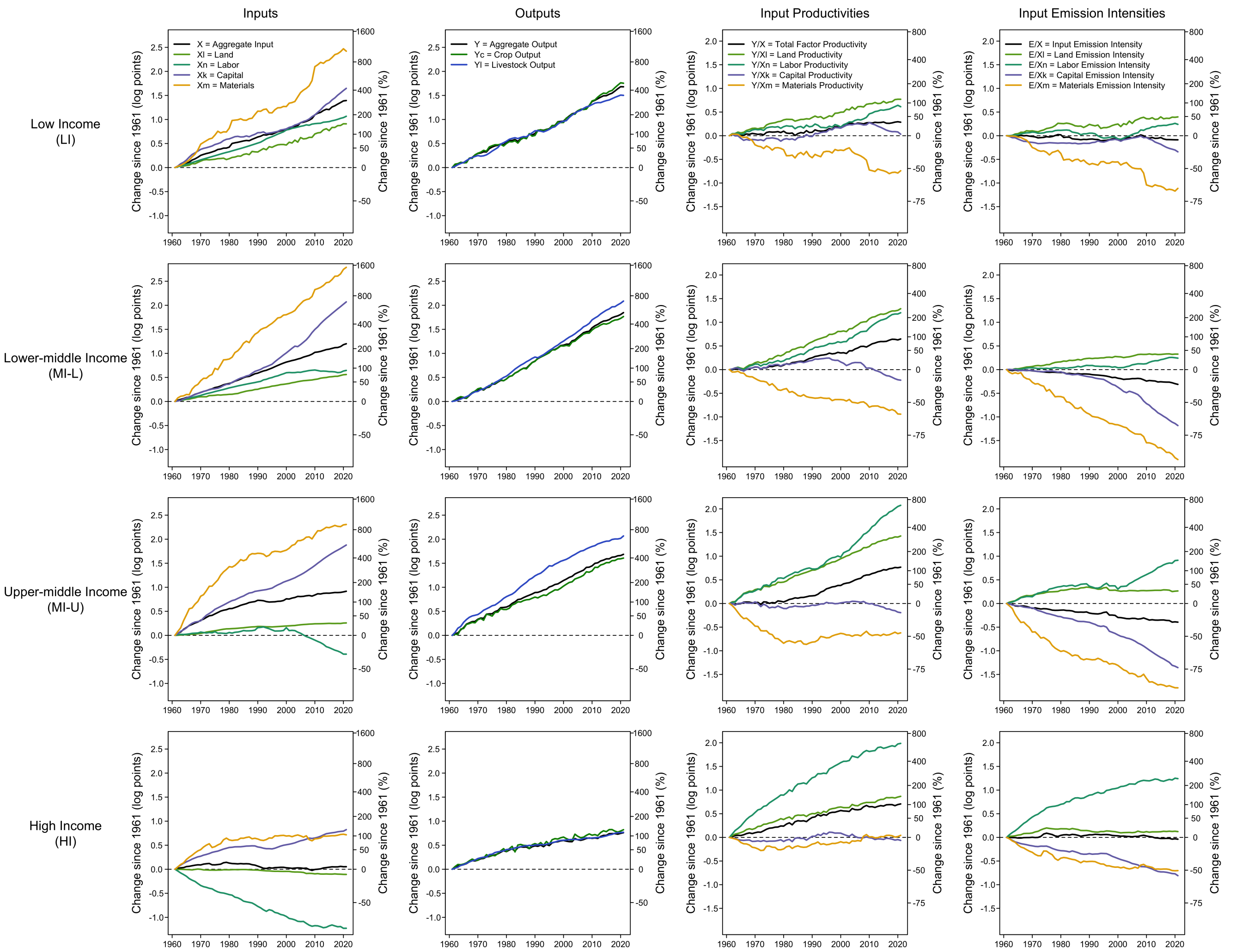

Figure S15: Relative changes in various indicators since 1961 (3 greenhouse gas categories for the 4th column).

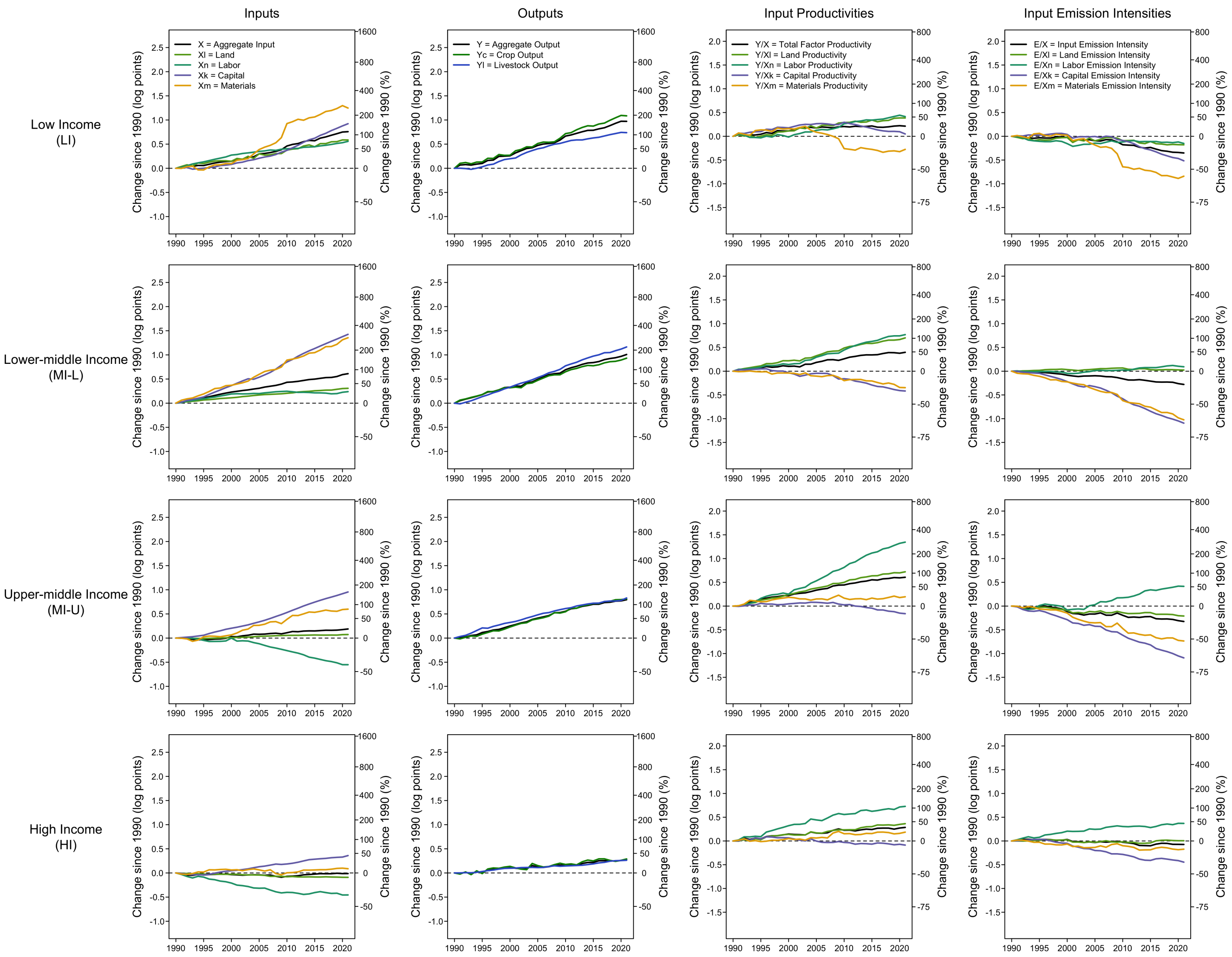

Figure S16: Relative changes in various indicators since 1990 (7 greenhouse gas categories for the 4th column).

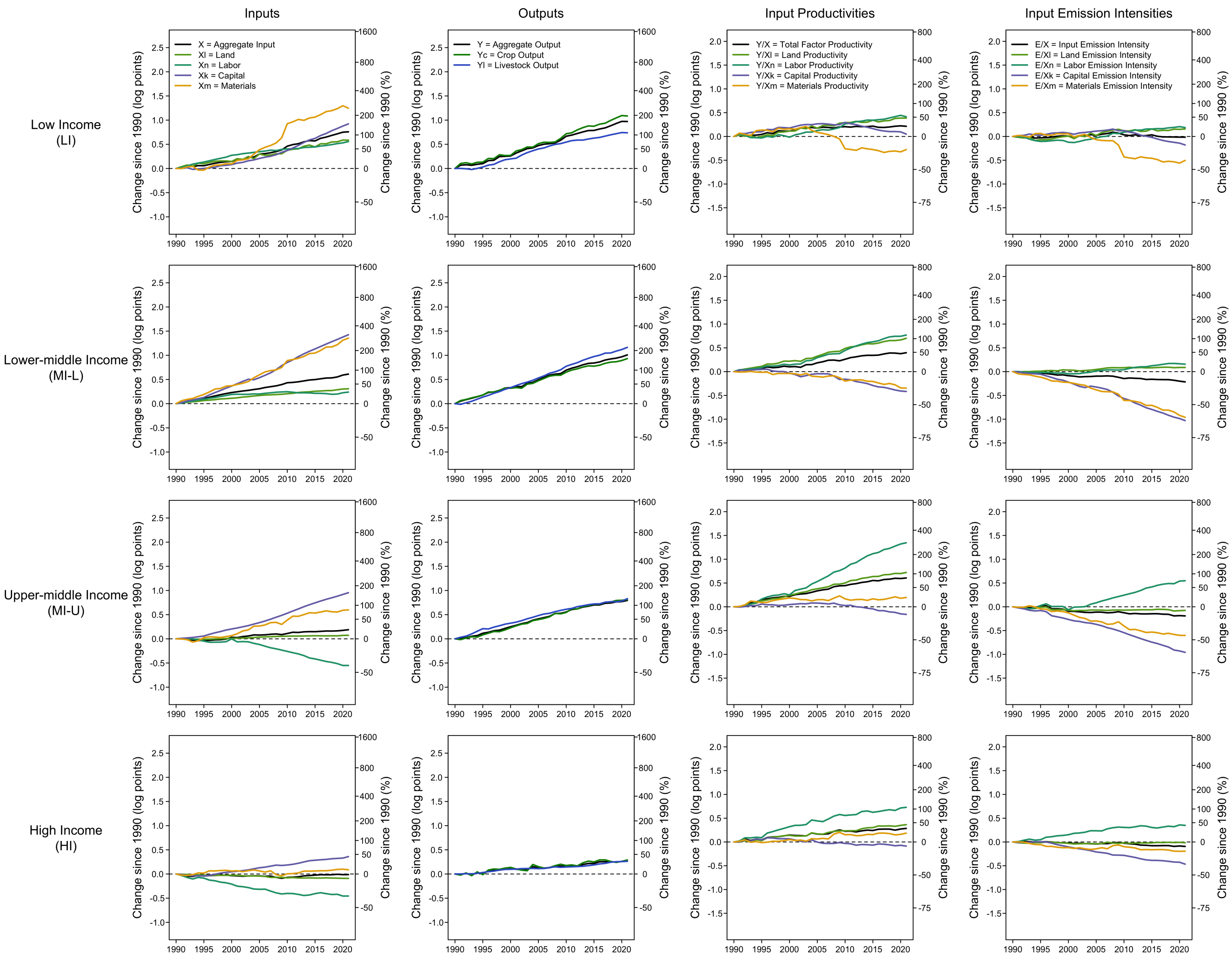

Figure S17: Relative changes in various indicators since 1990 (3 greenhouse gas categories for the 4th column).

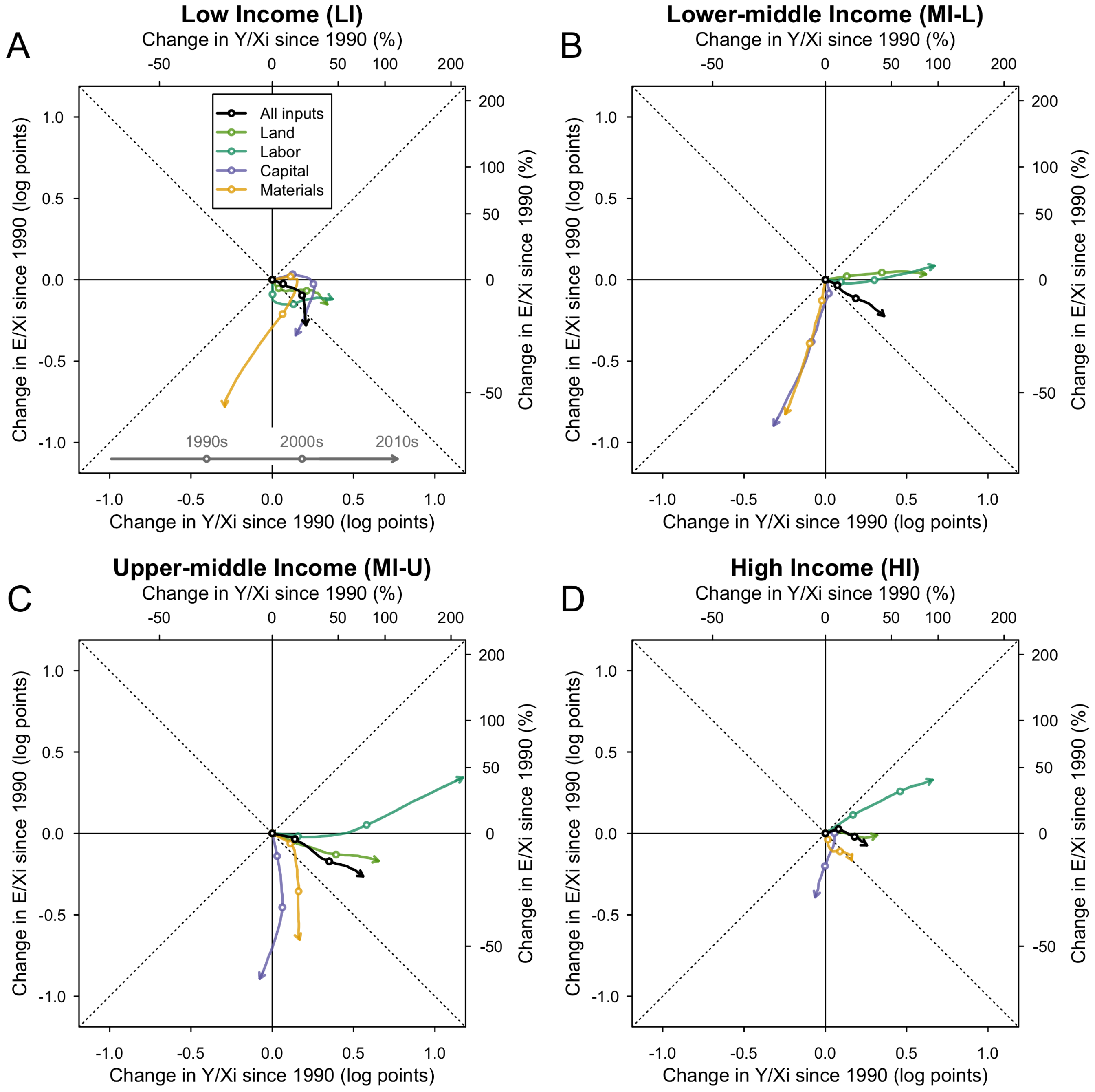

Figure S18: Evolution of partial input emission intensities and factor productivities by income group since 1990 (7 greenhouse gas categories).

This is analogous to Fig. 5 but considers changes since 1990 and for 7 GHG emission categories. Each panel showcases the evolution of the overall input emission intensities and total factor productivity shown in Fig. 3D in black. We then represent the partial emission intensities (E/X_i) and partial factor productivities (Y/X_i) in colored lines. (**A**) Low-income. (**B**) Lower-middle income. (**C**) Upper-middle Income. (**D**) High Income.

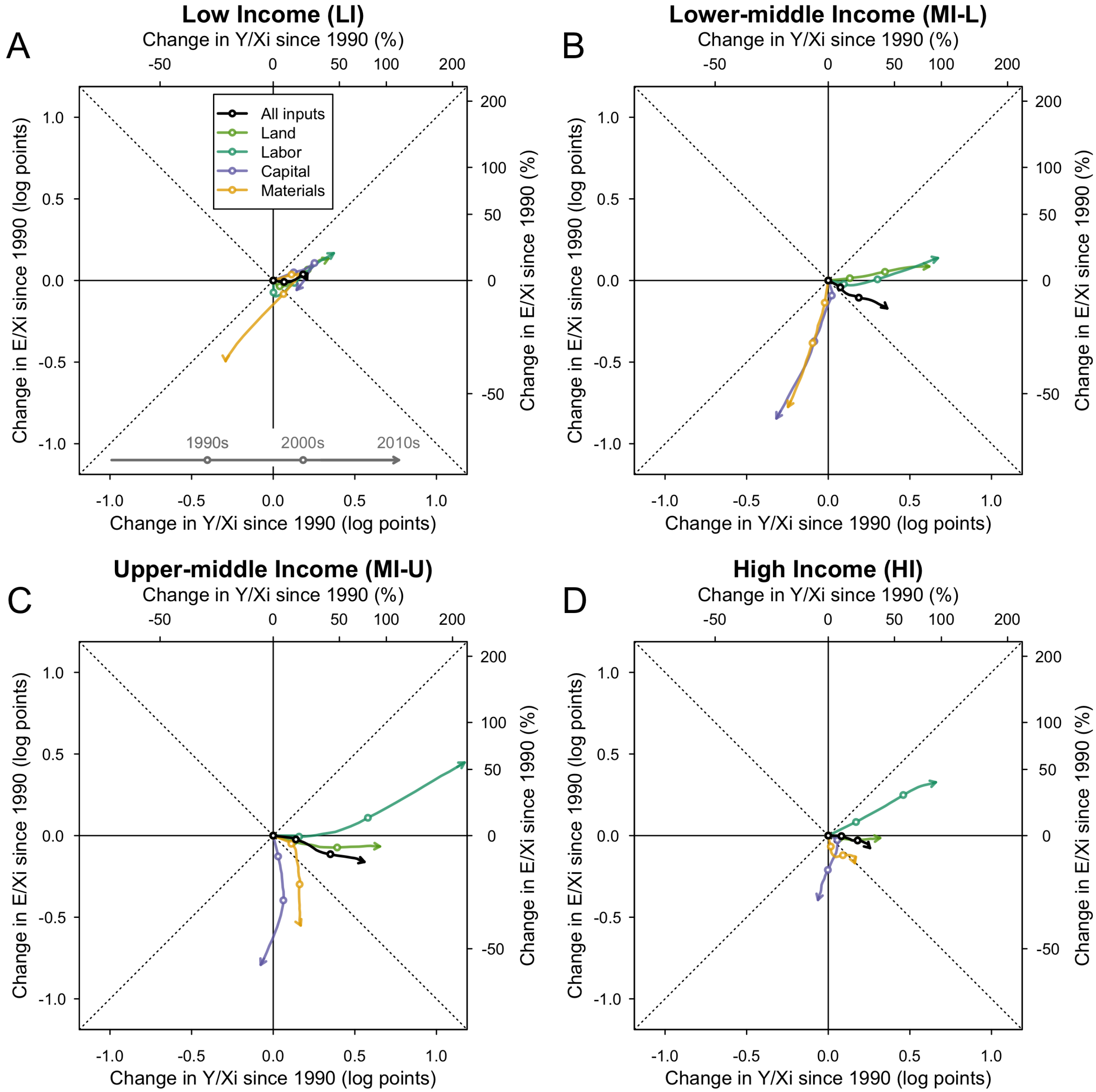

Figure S19: Evolution of partial input emission intensities and factor productivities by income group since 1990 (3 greenhouse gas categories).

This is analogous to Fig. 5 but considers changes since 1990 and for the same 3 GHG emission categories used in the main text. Each panel showcases the evolution of the overall input emission intensities and total factor productivity shown in Fig. 3D in black. We then represent the partial emission intensities (E/X_i) and partial factor productivities (Y/X_i) in colored lines. (**A**) Low-income. (**B**) Lower-middle income. (**C**) Upper-middle Income. (**D**) High Income.

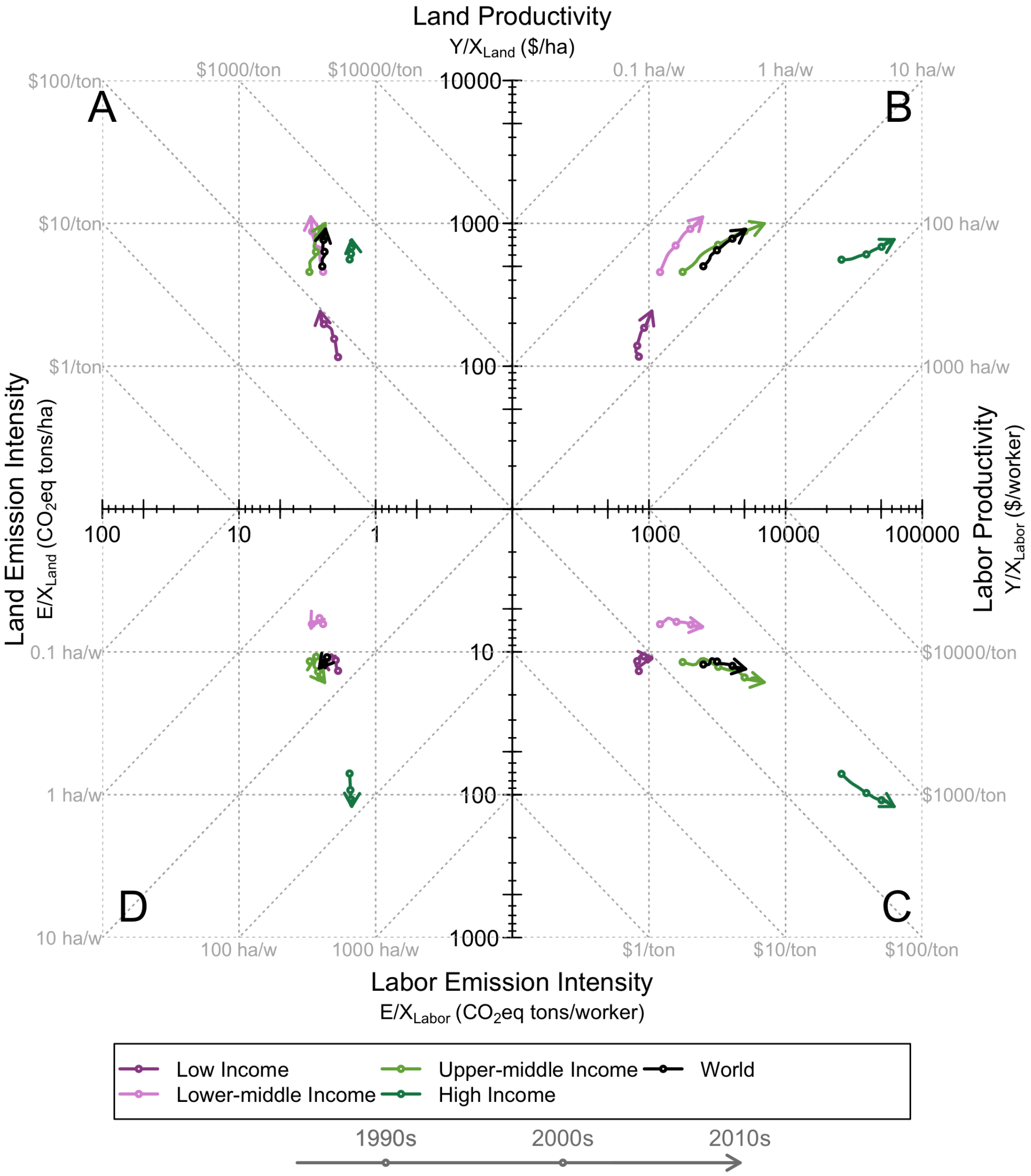

Figure S20: Level changes in partial input emission intensities and factor productivities for land and labor since 1990 (7 greenhouse gas categories).

This is analogous to Fig. 6 but considers changes since 1990 and for 7 GHG emission categories. Each panel showcases the evolution, in levels, of partial input emission intensities (E/X_i) and/or partial factor productivities (Y/X_i) by income group, where the subscript i denotes the input. All axes on log scale. Diagonal dotted lines correspond to fixed ratios in inputs (land and labor) or outputs (marketed output and emissions). (**A**) Land productivity Y/X_{Land}, versus Land emission intensity, E/X_{Land}. (**B**) Land productivity Y/X_{Land}, versus Labor productivity Y/X_{Labor}. (**C**) Labor emission intensity E/X_{Labor}, versus Labor productivity Y/X_{Labor}. (**D**) Labor emission intensity E/X_{Labor}, versus Land emission intensity E/X_{Land}.

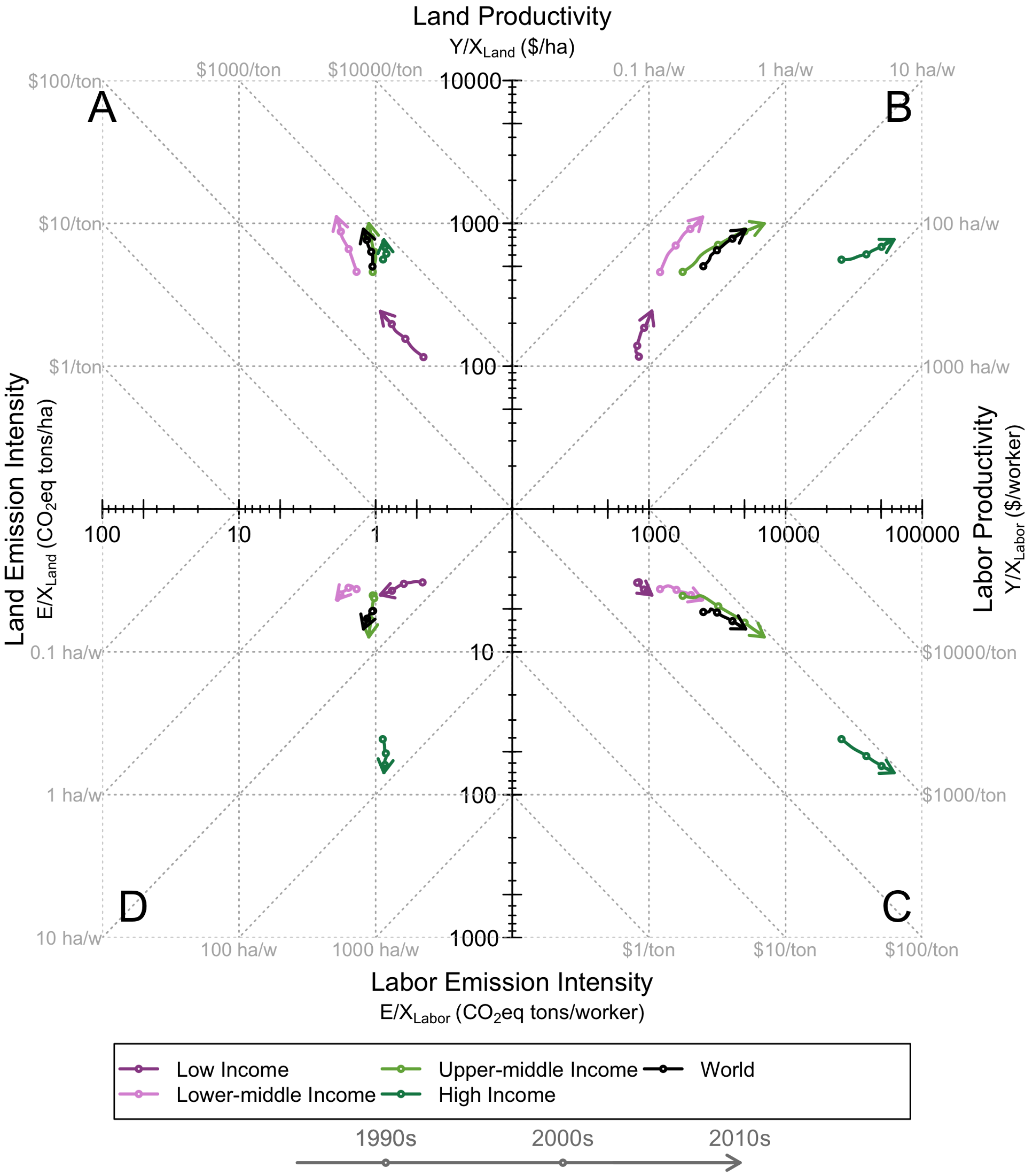

Figure S21: Level changes in partial input emission intensities and factor productivities for land and labor since 1990 (3 greenhouse gas categories).

This is analogous to Fig. 6 but considers changes since 1990 and for the same 3 GHG emission categories used in the main text. Each panel showcases the evolution, in levels, of partial input emission intensities (E/X_i) and/or partial factor productivities (Y/X_i) by income group, where the subscript i denotes the input. All axes on log scale. Diagonal dotted lines correspond to fixed ratios in inputs (land and labor) or outputs (marketed output and emissions). (**A**) Land productivity Y/X_{Land}, versus Land emission intensity, E/X_{Land}. (**B**) Land productivity Y/X_{Land}, versus Labor productivity Y/X_{Labor}. (**C**) Labor emission intensity E/X_{Labor}, versus Labor productivity Y/X_{Labor}. (**D**) Labor emission intensity E/X_{Labor}, versus Land emission intensity E/X_{Land}.

Table S1: Input cost shares used by USDA ERS for deriving TFP estimates.

Cost shares for each country or region sum to 1 by definition. Years in 1960s correspond to 1961-1970, and so forth for the other decades.

	Labor							Land						
Region	1960s	1970s	1980s	1990s	2000s	2010s	2020s	1960s	1970s	1980s	1990s	2000s	2010s	2020s
World	0.286	0.286	0.285	0.296	0.300	0.308	0.311	0.232	0.219	0.199	0.225	0.232	0.236	0.239
HI	0.248	0.231	0.185	0.220	0.233	0.220	0.220	0.166	0.176	0.182	0.183	0.153	0.167	0.171
LI	0.247	0.249	0.246	0.232	0.231	0.238	0.240	0.308	0.303	0.304	0.324	0.330	0.323	0.321
MI-L	0.337	0.370	0.445	0.423	0.391	0.397	0.400	0.307	0.243	0.217	0.247	0.302	0.298	0.296
MI-U	0.326	0.330	0.332	0.307	0.308	0.317	0.317	0.293	0.262	0.201	0.245	0.245	0.237	0.237
USA	0.232	0.180	0.163	0.209	0.237	0.175	0.173	0.168	0.211	0.148	0.133	0.090	0.162	0.172
CHN	0.443	0.396	0.413	0.333	0.333	0.333	0.333	0.240	0.194	0.169	0.247	0.258	0.258	0.258
IND	0.406	0.419	0.564	0.554	0.505	0.505	0.505	0.314	0.210	0.173	0.181	0.267	0.267	0.267
BRA	0.434	0.434	0.443	0.415	0.373	0.373	0.373	0.342	0.342	0.159	0.115	0.083	0.083	0.083
SVU	0.112	0.113	0.113	0.182	0.210	0.291	0.291	0.253	0.251	0.250	0.247	0.222	0.113	0.114

	Capital							Materials						
Region	1960s	1970s	1980s	1990s	2000s	2010s	2020s	1960s	1970s	1980s	1990s	2000s	2010s	2020s
World	0.207	0.217	0.238	0.233	0.227	0.221	0.220	0.275	0.279	0.278	0.247	0.241	0.235	0.230
HI	0.196	0.196	0.236	0.234	0.239	0.223	0.220	0.390	0.398	0.396	0.363	0.374	0.390	0.389
LI	0.290	0.291	0.289	0.299	0.295	0.307	0.312	0.155	0.157	0.160	0.146	0.145	0.131	0.128
MI-L	0.241	0.256	0.191	0.197	0.168	0.170	0.171	0.115	0.131	0.147	0.134	0.140	0.135	0.134
MI-U	0.199	0.221	0.259	0.244	0.241	0.240	0.240	0.182	0.187	0.208	0.205	0.207	0.206	0.206
USA	0.268	0.251	0.363	0.343	0.333	0.286	0.276	0.332	0.358	0.326	0.316	0.339	0.376	0.380
CHN	0.211	0.279	0.286	0.256	0.264	0.264	0.264	0.106	0.131	0.133	0.163	0.144	0.144	0.144
IND	0.216	0.279	0.147	0.158	0.117	0.117	0.117	0.064	0.092	0.116	0.107	0.111	0.111	0.111
BRA	0.167	0.167	0.200	0.247	0.214	0.214	0.214	0.057	0.057	0.198	0.224	0.330	0.330	0.330
SVU	0.221	0.219	0.218	0.218	0.269	0.255	0.256	0.415	0.413	0.412	0.354	0.299	0.340	0.339

Table S2: Growth of various indicators over 1961-2021 based on 3 greenhouse gas (GHG) emission subcategories.

Y represents total agricultural output; X represents for aggregate agricultural inputs; E represents greenhouse gas emissions; E/Y represents output emission intensity; E/X represents input emission intensity; Y/X represents Total Factor Productivity (TFP). The 3 GHG subcategories correspond to: emissions from crops, emissions from livestock and synthetic fertilizers (available since 1961; see Fig. S1).

	Annual growth rate (%/year)						Total growth (%)					
Region	Y	X	E	E/Y	E/X	Y/X	Y	X	E	E/Y	E/X	Y/X
World	2.203	0.962	0.622	-1.547	-0.337	1.229	269.711	77.654	45.082	-60.758	-18.334	108.108
HI	1.274	0.088	0.024	-1.234	-0.063	1.186	113.793	5.400	1.469	-52.539	-3.730	102.839
LI	2.839	2.350	2.202	-0.620	-0.145	0.478	436.391	303.039	269.412	-31.130	-8.343	33.087
MI-L	3.127	2.020	1.491	-1.587	-0.519	1.085	534.387	232.009	142.990	-61.697	-26.812	91.075
MI-U	2.848	1.540	0.876	-1.918	-0.655	1.288	439.214	150.206	68.711	-68.712	-32.571	115.508
USA	1.497	0.291	0.205	-1.273	-0.086	1.202	143.896	19.073	13.105	-53.626	-5.013	104.828
CHN	3.860	2.049	1.252	-2.511	-0.781	1.775	870.472	237.773	110.988	-78.259	-37.536	187.315
IND	2.908	1.604	1.036	-1.819	-0.559	1.283	458.324	159.807	85.586	-66.760	-28.568	114.900
BRA	3.473	1.685	2.304	-1.130	0.608	1.759	675.726	172.557	292.153	-49.447	43.879	184.611
SVU	1.039	0.413	-0.404	-1.428	-0.813	0.624	85.908	28.026	-21.573	-57.814	-38.741	45.212
EUN	0.708	-0.326	-0.241	-0.942	0.085	1.037	52.672	-17.810	-13.487	-43.334	5.261	85.756

Table S3: Growth of various indicators over 1990-2021 based on 7 greenhouse gas (GHG) emission subcategories.

Y represents total agricultural output; X represents for aggregate agricultural inputs; E represents greenhouse gas emissions; E/Y represents output emission intensity; E/X represents input emission intensity; Y/X represents Total Factor Productivity (TFP). The 7 GHG subcategories correspond to: emissions from crops, emissions from livestock and synthetic fertilizers (available since 1961) as well as drained organic soils, on-farm energy, savannah fires and land use change (available since 1990). See Fig. S1.

	Annual growth rate (%/year)						Total growth (%)					
Region	Y	X	E	E/Y	E/X	Y/X	Y	X	E	E/Y	E/X	Y/X
World	2.073	0.620	-0.074	-2.103	-0.690	1.444	88.911	21.137	-2.260	-48.261	-19.315	55.948
HI	0.885	-0.040	-0.276	-1.151	-0.236	0.926	31.425	-1.242	-8.207	-30.156	-7.052	33.079
LI	3.185	2.476	1.321	-1.806	-1.127	0.691	164.270	113.440	50.212	-43.160	-29.623	23.815
MI-L	3.306	1.990	1.076	-2.159	-0.897	1.290	174.073	84.207	39.336	-49.161	-24.359	48.786
MI-U	2.597	0.609	-0.438	-2.958	-1.041	1.976	121.370	20.709	-12.724	-60.575	-27.697	83.392
USA	1.345	0.569	0.072	-1.256	-0.493	0.772	51.318	19.213	2.270	-32.414	-14.213	26.931
CHN	3.500	0.868	0.416	-2.979	-0.448	2.610	190.501	30.714	13.742	-60.846	-12.984	122.242
IND	3.152	1.626	1.183	-1.909	-0.436	1.501	161.680	64.877	43.989	-44.975	-12.669	58.713
BRA	3.489	1.285	-1.119	-4.452	-2.374	2.175	189.517	48.568	-29.446	-75.631	-52.511	94.872
SVU	0.208	-1.353	-2.139	-2.343	-0.797	1.583	6.656	-34.453	-48.850	-52.042	-21.964	62.717
EUN	0.118	-0.817	-0.700	-0.817	0.118	0.943	3.709	-22.467	-19.569	-22.445	3.738	33.760

Table S4: Growth of various indicators over 1990-2021 based on 3 greenhouse gas (GHG) emission subcategories.

Y represents total agricultural output; X represents for aggregate agricultural inputs; E represents greenhouse gas emissions; E/Y represents output emission intensity; E/X represents input emission intensity; Y/X represents Total Factor Productivity (TFP). The 3 GHG subcategories correspond to: emissions from crops, emissions from livestock and synthetic fertilizers (available since 1961; see Fig. S1). Naturally, this table only differs from the previous one for columns that relate to GHG emissions (i.e. E, E/Y and E/X).

	Annual growth rate (%/year)						Total growth (%)					
Region	Y	X	E	E/Y	E/X	Y/X	Y	X	E	E/Y	E/X	Y/X
World	2.073	0.620	0.128	-1.906	-0.490	1.444	88.911	21.137	4.038	-44.927	-14.116	55.948
HI	0.885	-0.040	-0.341	-1.216	-0.301	0.926	31.425	-1.242	-10.061	-31.566	-8.930	33.079
LI	3.185	2.476	2.427	-0.734	-0.047	0.691	164.270	113.440	110.330	-20.411	-1.457	23.815
MI-L	3.306	1.990	1.283	-1.958	-0.693	1.290	174.073	84.207	48.467	-45.830	-19.402	48.786
MI-U	2.597	0.609	-0.010	-2.541	-0.616	1.976	121.370	20.709	-0.325	-54.973	-17.425	83.392
USA	1.345	0.569	0.167	-1.162	-0.399	0.772	51.318	19.213	5.310	-30.405	-11.663	26.931
CHN	3.500	0.868	0.193	-3.195	-0.669	2.610	190.501	30.714	6.151	-63.459	-18.791	122.242
IND	3.152	1.626	0.861	-2.220	-0.752	1.501	161.680	64.877	30.461	-50.145	-20.873	58.713
BRA	3.489	1.285	1.469	-1.952	0.181	2.175	189.517	48.568	57.151	-45.720	5.777	94.872
SVU	0.208	-1.353	-2.105	-2.308	-0.762	1.583	6.656	-34.453	-48.291	-51.518	-21.111	62.717
EUN	0.118	-0.817	-0.975	-1.092	-0.159	0.943	3.709	-22.467	-26.206	-28.845	-4.822	33.760
